# 2050: An Arthroplasty Odyssey †

**DOI:** 10.3390/healthcare13212730

**Published:** 2025-10-28

**Authors:** Eloy del Río

**Affiliations:** Independent Researcher, 11520 Cádiz, Spain; eloy.delrio@uca.es

**Keywords:** osteoarthritis epidemic, joint replacement surgery, critical material scarcity, supply chain risk management, health equity and disparities, health economics, preventive health/sustainable green policy, climate change/crisis/emergency, net-zero emissions/decarbonization, energy transition/renewable energy integration/electrification

## Abstract

**Highlights:**

**What are the main findings?**

- Advocates the inclusion of critical raw material management and supply chain resilience in health policy planning to address imminent scarcities and ensure the sustainability of essential medical services, such as arthroplasty, potentially impacted by Net Zero by 2050.

**What is the implication of the main finding?**

- Interdisciplinary health policy frameworks that incorporate insights from healthcare, environmental science, and supply chain management are essential to effectively address the complex challenges of achieving sustainable, equitable, and ethical arthroplasty practices during the escalating osteoarthritis epidemic.
- Highlights the need for preventive health policies that promote early detection and treatment of osteoarthritis, aiming to decrease long-term reliance on joint replacement surgeries, thereby reducing future demand pressures and enhancing patient outcomes.

**Abstract:**

Drawing inspiration from Stanley Kubrick’s iconic science fiction masterpiece, this study posits that the future of joint health is not confined to a singular trajectory but is instead shaped by our collective efforts towards pioneering initiatives that transcend present-day boundaries. From its inception to the horizon of 2050, the trajectory of arthroplasty presents a compelling narrative of medical innovation, socioeconomic challenges, and sustainability pursuits. This Perspective addresses the growing osteoarthritis epidemic, emphasizing the urgent need for prevention and early-intervention strategies to reduce disease progression in the context of imminent critical-raw-material scarcity and the transition to a carbon-free economy. This transition, aiming for Net Zero by 2050, may unintentionally lead to financial instabilities and healthcare disruptions—driven by supply-chain fragility and rising costs—and could thereby exacerbate inequities in access to elective joint replacement. The illustrative scenarios and conditional comparative trends presented here highlight potential co-occurring clinical, economic, and material risks under business-as-usual (BAU) assumptions. These multifaceted complexities warrant the development of coordinated strategies. By examining current trends and future challenges, this paper therefore calls for a holistic approach to the green transition that promotes multidisciplinary dialogue and policy alignment to ensure an ethical, equitable, and sustainable future for resilient arthroplasty services amid ongoing decarbonization initiatives.

## 1. The Dawn of the Arthroplasty Era

The arthroplasty era emerged against the backdrop of the crisis of osteoarthritis (OA), especially in the knees and hips, a leading cause of disability and one of the most prevalent chronic non-communicable diseases globally [1,2]. Transitioning from sporadic cases in the early 20th century to a significant public health concern today, the evolution of total hip arthroplasty (THA) and total knee arthroplasty (TKA) mirrors the growing epidemic of OA. In the United States, this condition is the primary cause of disability in 8.6 million American adults [3], while 7 million individuals live with hip or knee replacement [4]—approximately the population size of New York City. The advent of arthroplasty revolutionized end-stage OA management, shifting care from conservative treatment toward surgical interventions that markedly enhance quality of life (QoL). This shift was driven by the urgent need to address the OA burden, which impacts millions worldwide, including young adults [5]. With roughly 600 million individuals affected [6,7], the prevalence of OA increases with age, imposing significant challenges on both individuals and healthcare systems. Projected trends by the GBD 2021 Osteoarthritis Collaborators [7] suggest that nearly 1 billion individuals might face OA by 2050, with an increase in knee OA cases by 74.9% and hip OA cases by 78.6% from 2020 to 2050. Such a concerning increase in OA prevalence demands a refined understanding and development of forward-thinking, preventive, and multidisciplinary management strategies that extend beyond traditional surgical approaches to include non-surgical options.

Orthopedic arthroplasties are meticulously engineered devices designed to replace or reconstruct damaged joints, effectively restoring mobility and alleviating persistent movement-evoked pain (MEP) in patients with knee and/or hip OA [8]. The introduction of THA in the mid-20th century, celebrated as a major surgical breakthrough [9], set a precedent for total joint replacement (TJR), offering hope to those suffering from end-stage OA pain and guiding the development of TKA. The evolution of arthroplasty, characterized by advancements in surgical techniques, prosthetic design, and postoperative care, reflects a deeper understanding of the biomechanics and tribology of human and artificial joints. Since the first successful TJR, there has been a continuous surge in the number of these surgical procedures, driven by their remarkable short-term benefits despite the important costs involved [10,11,12]. With hospital expenditures for arthroplasty procedures reaching $7 billion in 2014 [8], the United States performs over 500,000 THAs and nearly 800,000 TKA annually [4]. Currently, expenses have increased significantly and continue to rise. As OA prevalence and demand for these surgeries increase [11,13,14,15,16,17], the sustainability of healthcare systems to accommodate this growing need has emerged as a paramount concern. The COVID-19 pandemic further exacerbated these challenges, exposing vulnerabilities in orthopedic supply chains and the healthcare infrastructure. Widespread cancellations of elective (non-emergency) surgeries led to unprecedented backlogs in joint replacement procedures and shortages or delays in implant components [18,19,20,21,22]. Recent analyses indicate that recovery from this COVID-19-induced joint replacement deficit is likely to be protracted, particularly in public healthcare systems such as those of England, Wales, and Northern Ireland, underscoring long-term implications for surgical capacity and patient outcomes [22]. These challenges underscore the critical importance of ensuring the availability, quality, and cost-effectiveness of surgical interventions and the implementation of preventative measures.

The Kellgren–Lawrence (KL) grading system remains the cornerstone of radiographic assessment in OA, offering a succinct five-tier scale to quantify structural joint degeneration on standardized weight-bearing radiographs (Table 1). Originally described in 1957, it comprises five grades: grade 0 (no radiographic features of OA), grade 1 (doubtful joint space narrowing and possible marginal osteophyte formation), grade 2 (definite osteophytes and possible joint space narrowing), grade 3 (multiple moderate osteophytes, definite joint space narrowing, some sclerosis, and possible bone end deformity), and grade 4 (large osteophytes, marked joint space narrowing, severe subchondral sclerosis, and definite bony deformity). Each conventional grade reflects progressive structural deterioration, thereby enabling standardized patient stratification in both clinical practice and research settings. Nevertheless, its semiquantitative nature limits its sensitivity to early cartilage loss and subtle subchondral changes, leading clinicians and researchers to complement it with advanced imaging modalities, such as MRI or CT, to achieve a more nuanced evaluation of joint pathology. Moreover, radiographic findings often demonstrate considerable inter-observer variability and inconsistencies in detecting osteoarthritic changes, which can lead to under- or overestimation of disease severity. Although symptomatic OA patients may demonstrate greater radiographic joint space narrowing (JSN), evidence suggests that radiographic severity alone does not fully predict surgical referral, with pain and functional limitations often exerting a stronger influence [23]. Consequently, the interpretation of KL grades should be integrated with standardized clinical assessments, validated patient-reported outcome measures, and, where appropriate, more sensitive or quantitative imaging techniques to better inform prognosis and therapeutic decision-making.

**Figure 1:**
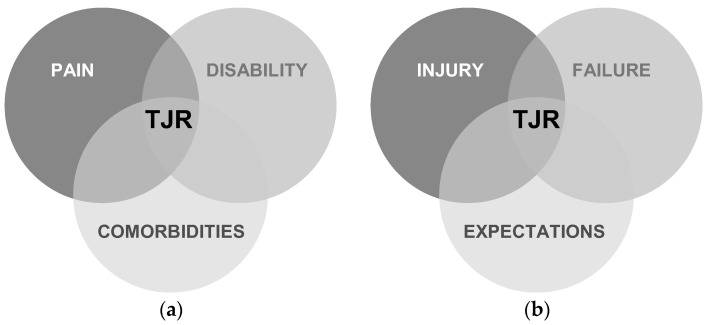
Conceptual illustration of multifactorial determinants driving demand for TJR in symptomatic end-stage OA across two age groups (young adults, 18–45 years; older adults, >65 years). The overlapping regions indicate combinations of clinical, functional, and contextual factors commonly associated with surgical referral. (**a**) In older adults, chronic MEP, progressive functional impairment, and OA-related comorbidities (e.g., diabetes, cardiovascular disease) collectively increase surgical demand, although radiographic JSN alone is not a reliable predictor of referral [23]. (**b**) In younger adults, demand is mainly driven by post-traumatic or overuse injury, limited response to conservative therapy, and high performance-related expectations for return to activity. This schematic illustrates contrasting population-level drivers to inform age-tailored preventive and treatment strategies.

In individuals with symptomatic OA, the decision to pursue TJR is driven by a complex interplay of clinical, functional, and psychosocial factors that vary markedly across the lifespan (Figure 1). Among older adults, escalating pain intensity and progressive functional disability remain the primary drivers of surgical referral, often compounded by multimorbidities such as cardiovascular disease, diabetes, and obesity, which both exacerbate joint symptoms and limit the efficacy of non-operative care [2,5]. Undoubtedly, arthroplasty continues to represent an effective treatment option for end-stage OA in this population, with evidence suggesting a survival benefit lasting up to 9–11 years postoperatively before an increase in mortality risk is observed [25]. Thus, individuals aged 65–85 years with moderate life expectancy are likely to derive the greatest net benefit from surgery [23]. By contrast, in middle-aged patients, candidacy for TJR is often driven by prior joint injury or cartilage damage unresponsive to conservative management (e.g., physiotherapy, intra-articular injections), coupled with distinct psychosocial expectations of rapid recovery, return to high-impact activities, and durable prosthetic performance, reflecting a strong desire to maintain occupational and recreational participation [26,27,28,29]. In this regard, the default expectation that “you replace the joint” when pain becomes intolerable is echoed in patient accounts [30]: *I was considering surgery because I believed that was how it was treated. That’s what you do when you have osteoarthritis and have pain, you replace your knee joint. That was the knowledge I had*. Such testimonies highlight how prevailing clinical practices, cultural narratives, and limited access to conservative care can funnel individuals toward elective surgery as the perceived first-line solution rather than as a last resort. However, when these expectations are not fully realized, decisional regret can arise, even in contexts where shared decision-making is employed, underscoring the importance of aligning surgical counseling with the lifestyle goals and risk profiles of patients [27]. For this younger group, the potential for higher long-term postoperative mortality further amplifies the need for a cautious risk–benefit assessment [27]. Taken together, these age-specific determinants underscore the imperative for a personalized, multidisciplinary approach in which elective surgical decision-making and postoperative goals are carefully matched to physiological reserve, comorbidity burden, lifestyle aspirations, and psychosocial priorities across different patient groups.

Reflecting on the dawn of the arthroplasty era, it is evident that the journey from its development to its current state is a testament to the relentless pursuit of medical excellence in confronting the challenges of knee and hip OA. Addressing this condition extends beyond alleviating immediate suffering and requires a broader vision of sustainable healthcare that aligns clinical practice and device manufacturing [31]. The International Energy Agency’s Net Zero Emissions (NZE) Scenario for 2050 provides the primary framework for the analysis presented in this study, ensuring that our recommendations align with global decarbonization goals [32]. Given the non-trivial carbon footprint associated with arthroplasty procedures—estimated to be on the order of a short transcontinental flight for a single operation [33]—the transition toward sustainable joint replacement practices is driven not only by visionary aspiration but also by the urgent need to address rising procedural demand, the scarcity of critical raw materials (CRMs), and the imperative of aligning healthcare with the Paris Agreement target of limiting global warming to well below 2 °C, and ideally to 1.5 °C, above pre-industrial levels [31,34]. Although essential for the production of orthopaedic implants, CRMs also play pivotal roles in clean-energy technologies, electronics, defense, and aerospace sectors [35,36,37]. Globally, thousands of metric tonnes of these minerals, especially cobalt and chromium, are consumed annually for total hip and knee arthroplasties, underscoring their strategic importance for public health and the sustainability of joint replacement programs.

Producers of joint implants with a high dependence on a small number of CRMs face mounting difficulties in securing reliable, cost-effective supplies, creating a strategic vulnerability for healthcare delivery. Recent geopolitical tensions, most notably the U.S.–China trade war and the associated reconfiguration of global supply chains, have exacerbated these uncertainties and risks by disrupting established trade flows and constraining access to critical minerals [38]. As China remains the dominant processor and exporter of several CRMs, export restrictions and tariff escalations have exposed the fragility of global medical device supply networks [39]. The resulting supply uncertainty and price volatility extend beyond the energy and electronics sectors, directly impacting the healthcare industry’s ability to secure stable and affordable inputs for implant manufacturing [40]. Such geopolitical disruptions expose healthcare systems to systemic vulnerabilities, as the production and delivery of orthopaedic implants depend on globally distributed sources of critical elements.

The increasing demand for TKAs and THAs among the aging population, combined with the potential scarcity and price volatility of CRMs for modern orthopaedic arthroplasties, urgently calls for a paradigm shift towards more resilient supply chain risk management strategies [37,41,42,43,44,45]. This new direction demands not only continuous innovations in surgical techniques but also holistic adoption of preventive measures to safeguard the future of orthopaedic healthcare [46]. As we look back on the origins of arthroplasty, the journey ahead requires evolving our approach to joint health and shifting from reactive interventions to proactive prevention and self-management strategies. Such a shift provides opportunities to mitigate the risk of supply chain disruptions in critical metals required for manufacturing joint implants amidst the immediate challenges posed by the green energy transition—particularly the electrification via solar, wind, hydroelectric, and hydrogen technologies—aimed at achieving a Net Zero by 2050 [32].

## 2. The Golden Age of Arthroplasty

The era that we now refer to as the golden age is characterized by an unprecedented level of innovation and advancement in the field of TKA and THA, offering millions of adults a chance to improve mobility and a life free from the debilitating pain of OA [9,11,47]. This period has witnessed the refinement of surgical techniques, the advent of advanced prosthetic materials, and an expansion in the criteria for elective TKA and THA, together positioning these procedures among the most successful and cost-effective interventions in modern medicine [12]. Computer- and robot-assisted surgeries combined with highly durable orthopaedic materials, such as ceramics, advanced polyethylenes, and metal alloys, have enhanced the precision of knee and hip joint replacements and substantially extended prosthetic implant longevity [48,49,50,51]. These technological advancements have been paralleled by an evolution in perioperative care, including enhanced recovery protocols that have drastically reduced hospital stay and improved patient outcomes. Despite its widespread adoption across industrialized health systems, the surgery-centric REPLACE model (Reactive End-stage Prosthesis for Load-bearing Arthritic Cartilage Erosion) has institutionalized a predominantly intervention-focused paradigm to OA care, one that prioritizes surgically advanced disease while systematically marginalizing preventive, rehabilitative, and disease-modifying strategies. This shift has raised concerns regarding the sustainability of the model and its inability to address the full spectrum of OA progression and patient needs in the long term [6,14,17,52].

**Figure 2:**
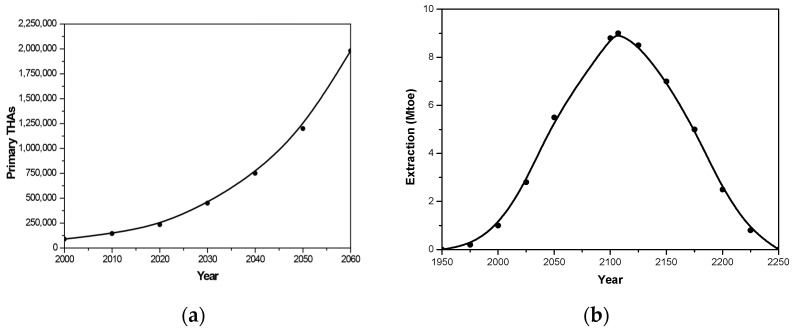
Comparative trajectories of (**a**) projected primary THA procedures in the U.S. Medicare population (2000–2060) and (**b**) illustrative Hubbert-style models of global chromium extraction (2000–2200), expressed in million tons of oil equivalent (Mtoe). Panel (a) reproduces the observed Centers for Medicare & Medicaid Services (CMS) data (2000–2019) and the point forecasts (2020–2060) from the log-linear (exponential growth) model by Schwartz et al. [15]; the 80% and 95% forecast intervals shown in the original publication are omitted here for clarity. Panel (b) adapts resource depletion parameterizations from ref. [53], depicting median illustrative trajectories within the uncertainty ranges detailed in [53]. Hubbert-style and long-range supply projections are scenario tools sensitive to resource estimates, by-product behaviour, demand shifts, substitution, recycling, and policy interventions and are therefore presented as provisional scenarios rather than deterministic forecasts. The two panels are presented as heuristic, comparative illustrations intended to highlight potential co-occurring trends in arthroplasty demand and critical material availability under business-as-usual (BAU) assumptions. They are not designed to imply direct causality or serve as predictive models but rather to provide a conceptual framework for examining systemic interdependencies and long-term sustainability challenges in joint replacement planning. All quantitative interpretations are conditional on the uncertainty bounds and parameter assumptions described in Appendix A and the cited sources.

While the golden age of arthroplasty has brought undeniable advancements, its success is increasingly overshadowed by significant challenges inherent to a model overly reliant on end-stage surgical intervention. In the United States, more than a million hips and knees are replaced each year—equivalent to one arthroplasty every half a minute—underscoring the immense and growing demand for joint replacement [4]. As shown in Figure 2, this demand for TKAs and THAs has risen exponentially, driven by an aging population, an increasing prevalence of obesity, and higher patient expectations for maintaining an active lifestyle [15]. Projections suggest a 176% increase in THA procedures by 2040, escalating to 659% by 2060 [15], placing a substantial burden on healthcare systems and raising questions regarding the sustainability of high-quality arthroplasty services. At the same time, the growing incidence of early-onset OA in younger patients introduces further complications regarding prosthetic durability and the potential for subsequent revision surgeries [4,11,13,16]. More than half of individuals with symptomatic knee OA are younger than 65 years [5], and many will live for three or more decades with the disease, underscoring the likelihood of greater cumulative disability and escalating healthcare utilization in the coming decades.

Comparable upward trajectories have been observed beyond the United States, although important regional differences shape both the demand dynamics and policy implications [54,55]. In Australia, Ackerman et al. [17] projected that, under continued growth trends, the number of primary TKAs could rise by 276% and THAs by 208% by 2030, reflecting the combined pressures of aging, obesity, and increasing demand. In Europe, multiple national and regional joint registries document consistent increases in arthroplasty volumes while revealing variations in device mix, clinical practices, and financing arrangements across health systems [56]. For example, in Germany, Rupp et al. [57] projected that the strongest relative proportional increase in primary TKA demand is expected among patients aged 40–69 years, particularly those aged 40–49 years (+269%), whereas for THA, the greatest increase is anticipated in older adults aged 80–89 years (+71%) between 2016 and 2040. These age-stratified patterns underscore how demographic structure, rather than overall population growth, will drive procedure volumes in aging yet shrinking populations. In the United Kingdom, projections based on Clinical Practice Research Datalink (CPRD) data forecast a sustained increase in both primary and revision arthroplasty rates, with procedure volumes expected to nearly double by the mid-2030s, largely reflecting demographic aging and progressively lower thresholds for surgical intervention [14]. By 2060, Matharu et al. [58] projected that demand would rise disproportionately in older age groups, particularly among those aged ≥ 80 years, highlighting the pronounced impact of demographic shifts on future healthcare needs. Rapid expansion has also been observed in Asia. China is reporting large and increasing caseloads alongside shifting demographic profiles, whereas India is scaling joint registry infrastructure and capacity to accommodate the growing demand [59,60,61]. In contrast, many low- and middle-income countries (LMICs) continue to perform very low volumes of TJRs due to constrained surgical capacity, high out-of-pocket expenditures, and fragile procurement and supply chain systems [62], even though feasibility studies have demonstrated that safe, high-quality arthroplasty can be delivered in well-prepared tertiary and district settings [63]. These regional contrasts in baseline volumes, device portfolios, procurement modalities, and financing mechanisms imply that supply shock scenarios—such as CRM bottlenecks—will produce heterogeneous effects worldwide, underscoring the need for context-sensitive, regionally tailored policy responses.

Moreover, the burden of OA and access to care are not distributed equally. In the United States, Black, Hispanic, and lower-income patients are consistently less likely to receive joint replacements, even when controlling for disease severity [64,65,66,67,68]. Delayed treatment often leads to worse outcomes, more complications, and longer recovery times compared with early management. Rural populations also face significant access barriers, with many living far from surgical centers or lacking the insurance coverage required for timely intervention [65]. These disparities point to a systemic failure in resource allocation and structuring of care delivery. Addressing these issues requires a shift toward more inclusive and equitable policies, such as expanding Medicaid coverage for conservative OA management, investing in community health programs, and supporting workforce development to bring orthopaedic and rehabilitation services into underserved areas.

Policy- and system-level responses must also guard against forces that could perversely entrench inequities and misdirect scarce resources. The rising uptake of robot-assisted and other high-cost technologies—most prominent in North America—risks encouraging a form of techno-optimism that privileges device-centered solutions over scalable, population-level prevention and rehabilitation [69,70]. This uncritical confidence in technology now extends beyond hardware to powerful digital tools, including large language models (LLMs) and AI-driven clinical support, which, if implemented without rigorous evaluation, may exacerbate bias, inequity, and misdirected investment [71]. These concerns are further supported by heterogeneous and inconsistent clinical and economic evidence surrounding digital health applications (such as artificial intelligence) and high-cost surgical technologies. Comparative cohort data do not yet demonstrate a clear long-term patient-utility advantage for robotic-assisted TKA versus conventional techniques [51], and systematic reviews of economic analyses reveal a strong association between the financial conflicts of interest of authors, industry funding, and conclusions favorable to new technologies, raising concerns about bias in the evidence base that shapes reimbursement and procurement decisions [72]. Conventional TJR, particularly the direct anterior approach to THA, also exhibits a substantive early learning curve, commonly cited at approximately 100 cases [73,74], meaning that at typical surgeon caseloads, achieving proficiency may take up to five years. Registry reports and volume–outcome studies highlight the wide inter-surgeon variability in annual THA volume and associated outcomes [75]. These findings underscore that procedural innovation alone is insufficient to improve outcomes without proactive workforce planning to ensure timely surgeon competency and patient access as the population burden of OA increases. This need is amplified by projections indicating a 2% decline in the total supply of orthopaedic surgeons by 2036, alongside an expected 10% increase in demand, which could reduce workforce adequacy from 100% in 2021 to 89% by 2036, with shortages most pronounced in non-metropolitan areas [76]. According to recent estimates, the average annual caseload of TJRs per orthopaedic surgeon is projected to more than double by 2050 to meet the rising procedural demand, further exacerbating concerns about sustainability and surgeon well-being [77].

Importantly, procedural innovation alone will not guarantee improved outcomes; substantial proportions of TKA and THA recipients continue to report unmet expectations, persistent pain, or limited function (12–20% across recent cohorts and reviews), with higher BMI, comorbidity burden, and adverse socioeconomic factors predicting worse patient-reported outcomes [28,29,78,79]. These persistent gaps in outcomes reflect patient heterogeneity and the inherent limitations of current prostheses. In response, orthopaedic implants have been the focus of extensive investigation in recent years, with researchers, clinicians, and industry stakeholders striving to elucidate their biomechanical performance, biological interactions, and long-term clinical implications. Yet, as Harman et al. [26] cautioned over a decade ago, *patients’ expectations that have been raised by aggressive marketing campaigns will have to be tempered by the reality that, in the next years at least, we do not see an implant on the market that will allow impact loading or vigorous athletic activities without a compromise in implant longevity*. Given these unresolved challenges in patient outcomes, the projected surge in OA cases, combined with the declining surgeon workforce and increasing risk of burnout among orthopaedic providers, further emphasizes the urgency of expanding and supporting the surgical workforce to meet future demand [13,80]. To avoid exacerbating disparities and maximize population health, policy must pair expanded access to conservative care with measures that increase transparency (independent cost-effectiveness evaluations, conflict-of-interest management), prioritize preoperative optimization and long-term patient-reported outcome measures (PROMs), and condition procurement and reimbursement on demonstrable, equitable value rather than novelty alone.

Furthermore, implant testing and rehabilitation protocols have been traditionally calibrated to standard Western gait datasets, which risks limiting the external validity of devices and conservative care programs when applied to culturally diverse populations. A recent study documented culturally specific activities of daily living (ADLs), including prayer movements and regional sitting/yoga postures, that impose distinct kinematic patterns and loading demands on lower limb joints [81]. The omission of such biomechanical diversity from design and preclinical testing may reduce the ecological validity of prosthetic implants and conservative care programs for many populations, thus representing an additional important equity and safety concern that warrants integration into device evaluation, registry design, and the development of culturally adapted clinical guidelines, rehabilitation protocols, and post-market surveillance systems. This issue is highly relevant to regulatory decision-making because implants intended for adaptation to non-standard functional demands will typically attract closer scrutiny [82]. In such cases, they may represent novel indications or configurations, require demonstration of safety and performance under non-standard loads, and necessitate longer-term outcome data to establish net clinical benefit. Practically, developers and policymakers should anticipate the need for more stringent premarket data packages, prospective registry enrolment, robust post-market follow-up, phased roll-out or conditional approvals, and carefully designed informed consent processes for early recipients. Incorporating culturally representative motion-capture datasets (e.g., Mihcin et al. [81]) into preclinical test plans, finite element and bench testing protocols, and registry data frameworks can strengthen regulatory submissions and promote more equitable, evidence-based evaluation and post-market surveillance.

Beyond these clinical challenges, this era is characterized by an increasingly unsustainable trajectory driven by the growing demand for joint replacements— largely fueled by the widespread adoption of the REPLACE model—and the imminent threat of CRM scarcity [35,36,37,41,44,45,53,83,84,85,86]. Certainly, the reliance on critical metals such as cobalt, chromium, molybdenum, and titanium in prosthetic manufacturing further complicates this scenario. These elements, essential for the efficacy and longevity of joint implants [48,49,50], face potential scarcity driven by escalating consumption and geopolitical instability, raising concerns about long-term material sustainability, supply security, and, ultimately, the future availability and economic viability of arthroplasty procedures. This critical juncture demands collective reevaluation and decisive strategic action. Exacerbated by CRM scarcity, disruptions in arthroplasty services may increase reliance on pharmaceutical treatments to manage advanced OA. Farrow et al. [19] reported that pandemic-related surgical delays in hip and knee arthroplasty were associated with increased opioid prescriptions and prolonged patient morbidity, underscoring the clinical and psychosocial impact of supply disruptions within orthopaedics. The widespread use of opioids, corticosteroids, and nonsteroidal anti-inflammatory drugs poses significant risks to public health in individuals with persistent joint pain. This dependency could significantly aggravate the opioid crisis, potentially increasing illicit fentanyl exposure among younger OA patients and contributing to rises in addiction, overdoses, and preventable deaths [5,25,87,88]. Consequently, further disruptions to the supply chain or significant increases in orthopaedic material costs not only threaten the standard practice of arthroplasty but also raise the morbidity and mortality rates among patients with knee and/or hip OA, urgently requiring a coordinated, systemic response to address these interconnected challenges.

The logistical disruptions triggered by Brexit and the COVID-19 pandemic exposed systemic fragilities in the global medical device supply chain, emphasizing the strategic necessity of developing adaptable, regionally diversified sourcing frameworks [21,22,38,42,89,90,91,92]. Trade restrictions and external shocks, such as pandemic-induced factory closures, can abruptly disrupt material availability. In parallel, geopolitical conflicts affecting CRMs, exemplified by the Russia–Ukraine war, threaten to exacerbate shortages and further delay the delivery of medical-grade alloys to manufacturers in the arthroprosthetic industry [93]. Accordingly, international policy initiatives increasingly prioritize supply chain resilience [90,91,92]. For instance, the U.S. National Strategy for a Resilient Public Health Supply Chain establishes governance mechanisms to allocate scarce resources fairly [43]. Translating these governance principles to orthopaedic implants is critical; without proactive oversight, uncertainty-aware planning, and probabilistic risk assessment, manufacturers and healthcare providers may face delayed device availability and escalating costs driven by CRM bottlenecks [35,36,41,53,83,84,85,86].

## 3. Net Zero by 2050: Implications for Routine Arthroplasty Procedures

The International Energy Agency’s 2050 net-zero scenario anticipates a massive and rapid deployment of renewable energy sources, aiming to reduce reliance on oil, gas, and coal [32]. This ambitious global commitment to achieve net-zero carbon emissions by mid-century has triggered unprecedented environmental awareness, urging all sectors to rethink their practices and contributions toward this collective goal. To limit the global temperature rise to 1.5 °C—the threshold beyond which the Intergovernmental Panel on Climate Change (IPCC) [34] warns of dramatically elevated risks of ecological crises, including more frequent extreme weather events, accelerated biodiversity declines, rising sea levels, pandemics and threats to food security—greenhouse gas emissions must be reduced to net zero around 2050. This raises a critical question: Might decarbonization and the transition to green technologies—by reshaping material supply chains and manufacturing priorities—unintentionally constrain access to routine joint replacements?

To situate implant manufacturing within the broader decarbonization agenda, it is important to recognize that Net Zero 2050 transition pathways substantially increase demand for CRMs—notably cobalt, chromium, and other transition metals—leading to intensified competition between the energy and medical sectors [44,45]. These critical elements, essential to most implant designs, have become central to contemporary debates on long-term sustainability and supply security. Cobalt–chromium alloys have been valued for decades for their strength, corrosion resistance, and wear characteristics, and remain the metallurgical gold standard for load-bearing joint prostheses [48,49,50]. The scarcity of these elements, driven by both eco-friendly goals and cross-sectoral competition, poses potential risks to the routine execution of TKA and THA surgeries (see Figure 2; Table 2). Hubbert-style assessments remain a useful first-order scenario tool for several non-fuel minerals but are sensitive to reserve estimates, by-product dynamics, and substitution/recycling trends [53]. Accordingly, the risks to implant supply and cost should be viewed as conditional scenarios that warrant proactive R&D (substitution and low-mass design), procurement strategies (pooled purchasing and life-cycle procurement), and enhanced post-market surveillance to monitor device longevity and material circularity.

Beyond the direct pressure on CRM availability, implant manufacturers operate within highly globalized, multi-tiered supply chains that introduce additional vulnerabilities. The medical device supply network sources components across multiple continents and exhibits high market concentration, complex regulatory overlay, and limited upstream transparency, characteristics that amplify the transmission of upstream shocks (raw material shortages, processing bottlenecks, transport interruptions) into downstream device availability and lead times [40]. These dynamics mean that even if metal availability is stabilized, disruptions in subcomponent processing, shipping and distribution, currency fluctuations, or supplier capacity can generate cascading delays and price shocks for completed prosthetic devices. These multi-factor supply chain drivers—not only raw-material costs but also labour, transportation and logistics, regulatory compliance, and market-allocation behaviours—therefore represent an important, and sometimes underappreciated, threat to steady access to orthopaedic implants [21].

Although the NZE initiative is undoubtedly crucial, it paradoxically exacerbates pressure on the availability of CRMs essential for contemporary technology, thereby questioning the future viability and economic efficiency of TKA and THA procedures. Even under current conditions, total joint arthroplasties constitute a major financial burden: in high-income countries, the average cost of a single hip or knee replacement, including hospital care, implant expenditure, and rehabilitation, often exceeds US $15,000–25,000 [95]. Despite efficiencies such as same-day discharge and streamlined care pathways, these surgical procedures remain prohibitively expensive, underscoring how additional pressures from CRM scarcity, supply chain disruptions, or price volatility could further compromise affordability and equitable access [37].

Dominated by China, the CRM market faces a labyrinth of geopolitical, economic, and environmental challenges that further complicate the availability and accessibility of these materials [39]. According to the International Energy Agency’s forecasts [32,44], the expected growth in CRM demand along with a decline in diesel production—essential for mineral extraction—will significantly impact mineral prices, as evidenced by the 115.2% increase in chromite ore costs from 2020 to 2024 [37]. Amid a 6 percent increase in the value of global chromium consumption, rising from US $846 million in 2023 to US $900 million in 2024, South Africa’s chromite production is challenged by escalating ore prices, the technical and safety complexities of deep-level extraction, rising labor costs, rail transport constraints, and an unreliable electricity supply.

As the demand for essential elements and raw materials surges, driven not only by the healthcare sector but also by critical players in the energy transition technologies, such as electric vehicles and renewables [35,41,53,83,84,85,86], competition for these finite resources is predicted to intensify. Even before reaching Hubbert’s peak, a considerable risk emerges when the cumulative demand for critical metals exceeds the extractable reserves and the technological capacity for extraction [53]. By 2050, demand may surpass mining reserves, highlighting severe supply challenges that could impact the standard practices of arthroplasty. Any supply chain disruption or significant increase in material costs could directly affect the affordability and timely delivery of joint replacement surgeries [96].

In the context of tightening supply and rising costs, the risk of “shock transmission” across supply chains becomes substantial. Nze-Ekpebie et al. [40] noted that medical device supply chains tend to be complex, multi-tiered, and lacking in full transparency, such that disruptions upstream (e.g., in raw material or subcomponent supply) can cascade downstream, sometimes unpredictably. For instance, a disruption in a subcomponent supplier (e.g., a metallurgy or processing facility) in one country can lead to delayed availability of finished implants elsewhere. Such cascading failures could introduce lead times and buffer stock pressures that are inadequately considered in the typical procurement planning in surgical centers. Indeed, the dual pressure of CRM scarcity and global supply-chain fragility means that even devices whose metal content is not marginal may become vulnerable to component or logistical bottlenecks [84]. In extreme scenarios, producers might delay deliveries or ration supply to higher-margin markets, placing lower-margin surgical centers at a greater risk of shortage (see Table 2 for details).

The pursuit of net-zero emissions by 2050 poses both formidable challenges and unparalleled opportunities for innovation and leadership in orthopedic implant design. As the healthcare and materials sectors confront these realities, the net-zero agenda emphasizes the need for adaptability in preserving the future of joint replacement. This imperative calls for an intensified attention to sustainable practices, exploration of alternative biomaterials, and integration of environmental considerations into surgical medicine to enhance the resilience of arthroplasty services in a decarbonizing world. Given the composition of many contemporary prostheses, typically composed of complex metal alloys [48,49,50], full cradle-to-cradle circularity for all components remains technically and economically challenging [36,41,53,83,97]. With finite mineral resources, the capacity of annual arthroplasty operations within healthcare systems will inevitably face constraints, potentially leading to systemic risks. Yet, partial recovery and targeted recycling are achievable and should be actively pursued alongside substitution and lightening strategies. Practical pathways include the increased use of ceramic bearings where clinically appropriate, adoption of highly cross-linked and oxidation-stabilized polyethylenes (UHMWPE/LDPE/LLDPE/HDPE) to reduce reliance on metal articulating surfaces, exploration of high-performance polymers (PEEK/PEKK) in selected non-load-bearing components, lattice and topology optimization to reduce titanium mass, and surface-engineering approaches that extend implant life and reduce wear [50,98,99]. Each option involves trade-offs in mechanics, regulatory evidence needs, and lifecycle impacts; however, together, they offer a portfolio of realistic mitigation strategies that can reduce metal dependence and improve sustainability. Instead of targeting absolute circularity, evidence supports a pragmatic, staged strategy—testing safe metal-recovery processes for explants, encouraging material substitution where feasible, and embedding design-for-recycling criteria within procurement and regulation—to reduce material intensity without compromising safety or performance [82].

Another urgent question emerges: will the anticipated reduction in surgical procedures arise from prohibitive costs, potentially exacerbating surgical disparities and health inequity [100,101,102,103,104], or will it result from a strategic decrease in arthroplasty interventions? The latter scenario would require a paradigm shift away from reliance on unsustainable palliative medicine towards a model emphasizing preventive care and effective conservative strategies. Such a forward-thinking approach, characterized by strategic planning and efficiency, may yield significant long-term economic and environmental advantages [105]. The following discussion aims to further examine this direction, shedding light on the viability of preventive, resource-conscious pathways that align with broader environmental and health sustainability goals [31]. By embedding sustainability at the core of implant design, procurement, and clinical practice, the arthroplasty field can navigate CRM scarcity, support NZE objectives, and ensure equitable access to joint replacement surgery in a rapidly decarbonizing world.

## 4. Beyond 2050: The Future of Arthroplasty—Continuity or Transcendence?

In Stanley Kubrick’s “2001: A Space Odyssey”, society faces divergent pathways that emphasize either preservation of current models or systemic transformation. This metaphor captures the dilemma confronting arthroplasty and joint health policy: persist in scaling reactive surgical responses to growing need, or reorient health systems toward preventive, integrative strategies that reduce future demand for joint replacement. Such a future requires orthopaedics, rheumatology, and a broad multidisciplinary coalition, including primary care, sports medicine, physiotherapy, biomechanics, radiology, public health and behavioral and social sciences, communication experts, health economics, and policy-level decision-makers, to fundamentally rethink how joint health is managed and to place proactive OA prevention and timely interventions at the center of practice [106,107,108]. The defining challenge for the coming decades should not be how to perform more replacements but how to make fewer of them necessary.

The rising wave of OA constitutes an urgent policy challenge, demanding both strengthened prevention strategies and expanded capacity for joint replacement. As healthcare systems prepare for this mounting burden, it is essential to interrogate the drivers that have brought us to this critical juncture and develop innovative, scalable approaches to mitigate the OA epidemic. Accordingly, we face a pivotal question: How did a condition once regarded as inevitable become an epidemic of missed opportunities, and how can research and clinical practice now pivot toward true prevention and early intervention? The contemporary understanding of OA has progressed from a simplistic “wear-and-tear” model to the recognition of a multifactorial disease encompassing mechanical, metabolic, and low-grade inflammatory processes [109,110,111,112,113]. Yet despite these conceptual advances, the prevalence of OA continues to rise—a paradox that highlights the pressing need to translate mechanistic insights into integrated clinical care, public-health interventions, and policy actions. Only by integrating life-course prevention with targeted clinical interventions can the trajectory of OA be altered, and future dependence on late-stage surgical solutions can be meaningfully reduced [114].

Over the past several decades, several initiatives have been introduced to mitigate these challenges. In 1999, the United Nations, in partnership with the World Health Organization (WHO), national and international organizations representing individuals with musculoskeletal disorders, and healthcare professionals worldwide, launched the Bone and Joint Decade 2000–2010 [115,116]. This international campaign sought to advance the prevention, diagnosis, and treatment of musculoskeletal disorders, with the explicit aim of improving the QoL and reducing the public health burden. Although the Bone and Joint Decade catalyzed important collaborative efforts in awareness, research, and care delivery, its preventive objectives, particularly for OA, were not realized at the population health level. Building on that momentum, the Osteoarthritis Research Society International (OARSI) submitted a critical report to the U.S. Food and Drug Administration in 2016 advocating for the recognition of OA as a serious disease, and the WHO designated 2021–2030 as the Decade of Healthy Ageing [117], thereby emphasizing the need to address conditions that erode functional capacity and QoL. Together, these initiatives highlight the significant public health challenges posed by OA and the need to develop innovative therapies and comprehensive management strategies. Nevertheless, epidemiological data reveals substantial increases in OA prevalence, incidence, disability, and demand for TJR since these efforts began, which therefore calls for more robust, early-stage, and structural prevention strategies [2,5,106,107,108,114,118,119,120,121,122].

The challenge of inertia in addressing the OA epidemic critically influences the trajectory of joint health management. Such inertia represents the cumulative effect of decades of increasing OA prevalence, a trend largely driven by aging populations, sedentary lifestyles, obesogenic environments, injury, and other contributing risk factors, both modifiable and non-modifiable [52,55,123,124,125]. As the epidemic advances, it appears to gain momentum, approaching a critical “point of no return”, where the disease burden and the demand for joint replacement surgeries risk overwhelming healthcare infrastructure worldwide [14,17]. Simultaneously, the persistent or increasing demand for surgery highlights a growing mismatch between the need for TKA/THA [11,13,15,16] and the availability of CRMs, such as chromium and cobalt. This reveals the risks associated with material limitations, emphasizing the challenges of sustaining arthroplasty practices during the depletion of critical metal reserves [35,36,37,41,53,83,85,86]. Such depletion can lead to increased costs of joint replacement procedures, further exacerbating inequalities and health disparities [103,104]. This scenario underscores the importance of reimagining OA management not merely as a collection of isolated cases but as a comprehensive public health challenge demanding collective, proactive efforts. Addressing this may include comprehensive public health initiatives targeting modifiable OA risk factors [52,123], healthcare policies that emphasize early intervention and nonsurgical management, and the exploration of alternative orthopaedic materials and methods for arthroplasty that reduce dependence on scarce resources. Efforts should encompass diversifying supply chains for prosthetic materials, investing in telemedicine and technological innovations to enhance care accessibility through both face-to-face and teleconsultations, and encouraging interdisciplinary partnerships to drive the development of innovative solutions [119,126,127,128,129,130,131,132,133].

As illustrated in Figure 2 and detailed in Table 2, the critical stage of arthroplasty evolution is marked not only by emerging challenges but also by opportunities for evidence-based policy responses. This phase, potentially occurring before 2050, represents a pivotal moment when targeted interventions can significantly influence the trajectory of the OA epidemic, mitigating its impact and alleviating the looming burden on healthcare resources. The inertia of the OA epidemic, though formidable, is not immutable; strategic preventive measures can decelerate its momentum, providing a chance to reorient towards a more sustainable and healthy future. Nonetheless, tipping points—potentially irreversible changes within the OA epidemic and their impact on arthroplasty demand—are critical [14,15,17]. Such tipping points underscore the moments when the combined effects of risk factors, disease prevalence, and healthcare system burdens escalate to a threshold that catalyzes a rapid and possibly irreversible change in the management of OA and how arthroplasty services are delivered [17]. This emphasizes the urgent need for timely and effective interventions to address the evolving interconnected global landscape of joint health management preemptively.

The critical stage highlights a narrow window of opportunity to halt the momentum of the OA epidemic and address its severe consequences amid its rapidly increasing trend (see Figure 2 and Table 2 for details). This calls for an integrated approach that combines public health initiatives, clinical innovation, and policy reforms focused on preventing OA and facilitating early intervention [2,3,5,6,11,52,106,107,108,114,122,123,134]. Strategies such as comprehensive public education on OA risk factors, promotion of community-based physical activity programs, and provision of nutritional guidance can significantly contribute to reducing the incidence and severity of this condition by targeting the earliest stages of joint degeneration. Often overlooked osteoarticular risk factors—including obesity, muscle weakness, joint laxity, injuries, trauma, fractures, meniscectomy, joint overuse, immobilization, history of knee/hip pain, genetic predispositions, congenital abnormalities, and joint misalignments—warrant timely diagnosis, intervention, and proactive management by rheumatologists and orthopaedic surgeons [5,46,52,134,135].

**Figure 3:**
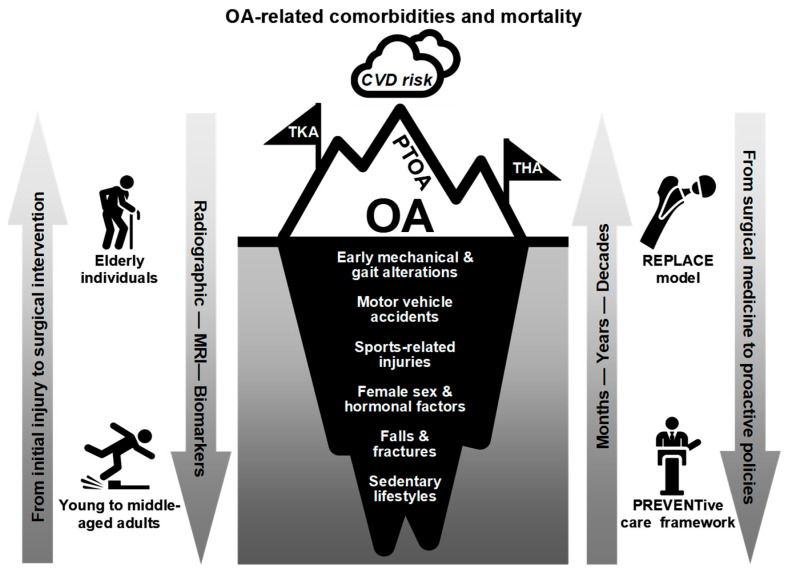
The OA iceberg: conceptual model of visible and hidden disease burden. Subclinical molecular and biomechanical alterations accumulate beneath the “waterline”, often preceding imaging-detectable changes. Symptomatic OA above the surface thus represents only a fraction of the total disease prevalence. The figure emphasizes the prolonged and variable natural history of OA, highlights the potential role of early molecular diagnostics, and supports the case for preventive interventions aimed at intercepting progression before irreversible structural joint damage occurs.

Bridging these preventive efforts with early therapeutic innovations is the next critical step. While lifestyle modifications and early risk management address upstream determinants [52,123], advances in non-surgical and regenerative interventions offer downstream opportunities to preserve joint integrity and delay disease progression. As depicted in Figure 3, utilizing advanced non-surgical techniques—particularly for asymptomatic, early-stage cartilage lesions—is crucial for preventing potentially avoidable post-traumatic osteoarthritis (PTOA), a clinically and biologically distinct subtype (pheno-endotype) of OA that arises following acute or chronic joint injury and is mediated by complex biomechanical, inflammatory, and reparative processes that accelerate cartilage breakdown and joint degeneration [136]. Table 3 summarizes this perspective by outlining the translational and policy priorities required to integrate early OA detection and intervention into clinical pathways and population-health strategies. Concurrently, advancements in early diagnosis and non-surgical management options, notably the coordinated application of pharmacological treatments and physical therapy, have the potential to delay or avoid joint replacement surgery [113,136,137,138,139,140,141,142]. This field is still far from mature. Policy reforms aimed at prioritizing evidence-based preventive healthcare [119], encouraging research and development in early OA interventions, and advocating the adoption of healthy lifestyles are crucial for effectively leveraging this opportunity. This helps ensure that the 2050 vision of arthroplasty becomes a reality, where the burden of OA is significantly alleviated and the resilience of healthcare systems is strengthened, paving the way for a more sustainable, equitable, and oriented toward a population-level health-focused future [31].

OA is often misunderstood to be a stable and easily manageable condition. However, it is characterized by significant variability in symptoms and progression [52,134]. Although it is necessary to develop effective management strategies for bad days, particularly for individuals experiencing difficulties with daily activities, OA should ideally be managed proactively before symptom onset. Achieving these objectives requires a profound shift in health policies to redefine current perceptions and understanding of OA. Despite its increasing prevalence, OA is often disregarded and marginalized in the public discourse, possibly because it does not directly cause death, although it significantly affects morbidity and QoL [52]. The considerable morbidity and mortality associated with this common condition are often underestimated due to its insidious progression and widespread misconceptions regarding its inevitability with aging [3,124,151,152]. This underscores the need for a nuanced health education approach that increases the perceived severity of OA to that of critical illnesses, such as heart disease, promoting early intervention strategies without resorting to fear tactics. Confronting the fatalistic view of OA as an unavoidable consequence of aging is crucial, as it delays timely medical engagement, reduces the chances of non-surgical interventions, and ultimately leads to a cascade of comorbidities exacerbating the condition [3,52,151]. This issue is exacerbated by systemic barriers to a timely diagnosis and access to care [153,154]. Additionally, it is imperative to address the prevailing techno-optimism associated with joint prostheses among patients with OA and, notably, among physicians who frequently underutilize conservative, non-surgical treatment options [51,96,152,155]. Despite high expectations, orthopaedic materials used in artificial joints still present significant challenges for tissue engineers because their mechanical properties fail to match the natural resilience and functionality of native human cartilage [48,49,50,69,70].

Addressing these challenges effectively requires comprehensive early intervention strategies for OA, including defining roles for information dissemination, developing accessible and engaging educational programs for joint health self-awareness, and encouraging proactive health behaviors at an early age [126,147,156,157,158]. A multidisciplinarity, interdisciplinarity and transdisciplinarity approach beyond medicine is essential for an effective public preventive education, paving the way for a future where OA management emphasizes innovation, environmental consciousness, and a deep commitment to enhancing patient well-being, with a particular focus on addressing the specific vulnerabilities associated with women [131,159,160]. A new model for understanding OA based on multiple phenotype/endotype-guided approaches has recently emerged to address disease heterogeneity and identify risk factors for the pathogenesis and progression of this complex joint condition [123]. The clinical heterogeneity of knee and hip OA poses a major challenge in treatment, requiring tailored therapeutic strategies to effectively address diverse phenotypes. Incorporating lifestyle factors into personalized diagnostics, patient stratification, predictive prognosis, and treatments tailored to specific OA phenotypes—including those associated with aging, physical inactivity, overweight/obesity, metabolic factors, post-traumatic injury, occupation, genetics, pain, and clinical, structural, and molecular characteristics—holds promise for achieving enhanced outcomes in the future [5,114,123,124,125]. This precision medicine strategy identifies distinct pathological processes and disease mechanisms in early stage OA, thereby enabling the timely application of tailored treatments. By integrating evidence-based pharmacological and non-pharmacological interventions, this approach has a significant potential for mitigating the need for prosthetic surgery.

For decades, OA has been characterized primarily as a “wear-and-tear” disease caused by cumulative mechanical overload, yet growing evidence suggests that habitual sedentary behavior may be as detrimental—or even more insidious—than excessive joint use [123]. Whittaker et al. [114] highlighted that the sharp rise in symptomatic hip and knee OA cannot be explained solely by increasing life expectancy or body mass but instead reflects post-industrial environmental changes that demand a life-course prevention approach. This interpretation is consistent with paleopathological evidence demonstrating that OA prevalence has escalated across historical populations and supports a shift in focus toward risk factors that emerged or intensified with industrialization [161]. Complementing this perspective, recent studies have proposed that thicker cartilage morphotypes, typically considered protective, can become metabolically vulnerable in sedentary contexts [125,162]. Without regular joint motion, nutrient transport into deep cartilage zones is compromised by stagnant synovial boundary layers, weakening proteoglycan metabolism, and predisposition to matrix breakdown. Simultaneously, inactivity leads to muscle weakness and impaired neuromuscular control, which concentrate joint loading and exacerbate the structural damage [123]. When these vulnerabilities converge with established risks, such as prior trauma, adiposity, or sex/gender-related factors, the combined effect may be synergistic, driving earlier and more severe disease expression [114]. These observations underscore the need to (1) incorporate cartilage morphotype (or “cartilotype”) and habitual activity patterns into risk stratification, (2) prioritize interventions that restore physiological joint loading through targeted activity, neuromuscular conditioning, and early post-injury rehabilitation, and (3) evaluate imaging and biochemical surrogates that capture both metabolic and mechanical aspects of joint health in prevention trials. Recognizing sedentary behavior as a modifiable, high-priority risk factor could therefore transform OA prevention, shifting the focus from late-stage treatment to early, mechanism-based interventions.

Building on this evidence, promoting active aging through comprehensive public health policies is essential to counteract the biological and social harms of inactivity and address the rising prevalence of knee and hip OA as the global population ages [7,124,163]. An integrated approach combining public awareness, education, and injury prevention can significantly reduce the burden of OA. The increasing incidence of PTOA among younger individuals, in particular, demands a multifaceted policy response to mitigate its public health impact [5,113,136,139,164,165,166]. Evidence from military and other physically demanding occupations reinforces these findings, showing a clear association between specific work tasks and acute articular cartilage injury [166]. Table 3 highlights the critical role of public health policy and management, advocating widespread campaigns to educate young and middle-aged adults on OA risk factors, early symptoms, and targeted preventive strategies. Injury prevention is particularly critical in sports and occupational settings [145,146,154]. Young women participating in sports face a 3–5 times higher risk of anterior cruciate ligament (ACL) injury than men, and factors such as genu valgum further elevate this risk and strongly predict PTOA [146,163,167]. Up to 87% of ACL injuries progress to PTOA [167]. Exercise-based prevention programs, early implemented in professional, amateur, and recreational sports, can substantially reduce the risk, with systematic reviews reporting a 52% decrease in female and 85% decrease in male athletes [168]. Additionally, minimizing activities that place excessive stress on the weight-bearing joints and avoiding trauma can significantly reduce the risk of PTOA [113,136,166]. A systematic review recently reported that individuals in highly physically demanding occupations, such as military service, face elevated incidence rates of acute articular cartilage tears (0.2–0.3 per 1000 person-years), yet underscored a paucity of data on specific occupational tasks and mechanisms alongside broader public health measures, highlighting an urgent need for targeted preventive measures in these work settings [166]. Collectively, minimizing high-stress joint activities, avoiding trauma, and implementing both sports- and occupation-specific preventive interventions can significantly reduce the risk of PTOA [113,136,166].

As shown in Table 3, concurrent measures, such as sports safety programs and enhanced workplace ergonomics, aim to lower injury rates, whereas road safety initiatives target the reduction in high-impact accidents that often lead to PTOA [164]. Advanced diagnostics and early detection tools, such as MRI and biomechanical assessments, ensure timely intervention [138,150,153]. Personalized rehabilitation programs designed to meet the unique needs of each patient based on factors such as age, joint condition, and functional goals, and incorporating modern technologies, play a crucial role in improving adherence, promoting effective recovery, and preventing chronic joint damage. Innovative regenerative therapies, including mesenchymal stem cell treatments and intra-articular injections of platelet-rich plasma (PRP), show promise for repairing joint tissues and delaying the onset of PTOA [169,170,171]. Stem cell therapy acts as a biological stimulator for cartilage regeneration by promoting chondrogenesis, mitigating cell senescence, suppressing osteoclast activity, enhancing subchondral bone remodeling, and repairing osteoarthritic cartilage to inhibit PTOA progression [169]. Conversely, PRP injection facilitates healing and reduces synovial inflammation [170]. A recent systematic review highlighted that the evidence base for these injectable and cell-based treatments is growing rapidly, especially for PRP and cell therapies, yet many existing trials remain short-term, lack standardized protocols, and often do not demonstrate clear disease-modifying effects [170]. Despite these advances, a universally accepted clinical protocol for managing knee and/or hip OA is still lacking. As highlighted by Malchau et al. [69], the stepwise introduction of innovative orthopedic therapies requires careful evaluation of efficacy and long-term safety, underscoring the uncertainties and challenges of translating preclinical promise into widespread clinical use and emphasizing the need for structured implementation alongside multidisciplinary strategies.

To achieve this objective, effective policy advocacy and robust health legislation are essential for securing sustained research funding, ensuring comprehensive access to healthcare, and facilitating integrated, multidisciplinary care pathways [82,131]. Furthermore, community engagement initiatives create supportive environments for joint health and economic incentives for healthcare providers and health educators to encourage adherence to best practices in non-pharmacological core OA management [172]. Collaborative efforts among healthcare providers, policymakers, and urban planners to create age-friendly environments [148], supported by public–private partnerships, enhance these strategies. Finally, policies promoting active aging and healthy longevity emphasize preventive healthcare, community support, and social inclusion to improve the QoL of older adults [119]. This holistic approach addresses immediate healthcare needs and aligns with broader public health goals, fostering a proactive stance toward OA prevention and management. In this context, interdisciplinary frameworks are essential, recognizing that clinical outcomes, environmental stewardship, and ethical sourcing are inherently interconnected and that only collective action can resolve these overlapping challenges [69,106,107,143]. By implementing these strategies alongside complementary interventions not covered in Table 3, we can effectively reduce the incidence and severity of OA and PTOA. This comprehensive approach supports healthier and more active lifestyles in the aging population, ultimately reducing the need for TJR surgeries.

Nonetheless, the greatest barrier remains the pervasive neglect of preventive and rehabilitative care services. Although arthroplasty incurs substantial costs, investments in preemptive measures are negligible. Evidence shows that targeted proactive interventions, such as structured muscle-strengthening regimens and modest weight-loss initiatives, can delay or avert the need for joint replacement in many cases. Australia’s National Osteoarthritis Strategy identifies obesity reduction, physical activity promotion, and joint injury prevention as its cornerstones but cautions that without scalable, policy-driven programs, both individuals and health systems will sustain avoidable OA burden [121]. Denmark’s GLA:D initiative has similarly produced significant pain relief and functional gains while deferring surgery [173]. Economic models reinforce these clinical outcomes: one Australian analysis estimated savings of roughly A$8000 per patient by postponing knee arthroplasty through a national exercise program [173]. Yet, rehabilitation remains critically under-resourced worldwide; as the WHO notes, “rehabilitation is often not a political priority” and is chronically underfunded [174]. Consequently, millions of individuals with musculoskeletal conditions lack access to physiotherapy, weight management support, or fall prevention programs, which are interventions proven to reduce surgical demand.

In addition, critical gaps in facilities, workforce capacity, and reimbursement structures across public health systems, including the U.S., prevent the effective scaling of preventive programs, representing a missed opportunity to reduce future arthroplasty demand and achieve substantial cost savings while improving patient outcomes [54,55,119,133]. Implementation evidence also underscores that scaling interventions depends critically on system-level enablers. For example, Walker et al. [175] used the RE-AIM framework in real-world OA settings and found that clinician engagement, patient recruitment strategies, and commissioning support were essential for sustainable scaling-up, as demonstrated in the ESCAPE-pain program, which achieved high adherence and sustained clinical benefits across multiple sites. Redirecting public funds toward these services would enhance the QoL of older adults, reduce disability, and relieve long-term financial pressures on health systems [121,173,176]. Consequently, these preventive efforts may deliver dual dividends—they foster healthier populations while diminishing surgical demand, thereby mitigating pressure on material supplies and enhancing equity.

**Figure 4:**
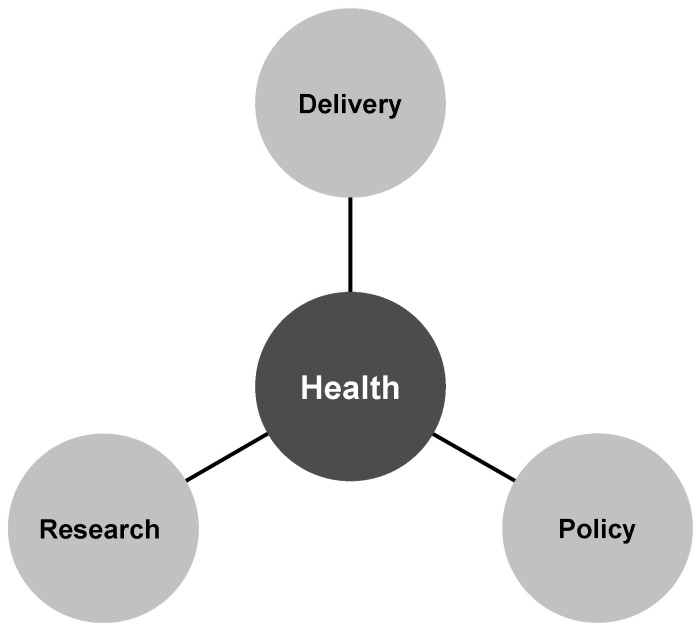
Conceptual schematic of the sustainable arthroplasty framework, positioning Health at the center of three interdependent pillars: research (evidence generation), policy (governance and financing), and delivery (service implementation). The model emphasizes the dynamic integration of these domains to advance clinical excellence, promote equity, and support sustainable environmental practices. Prevention across the life course (primary, secondary, and tertiary) is an explicit axis within this framework. Examples of objectives, target populations, and recommended outcome measures for each prevention level are summarized in Table 1.

Bridging prevention and intervention marks a defining test for 21st-century musculoskeletal medicine. The future of OA care depends not merely on treating disease earlier, but on transforming early management into a new frontier of joint preservation—where proactive prevention supplants mechanical replacement as the dominant paradigm. Building on this paradigm shift, Figure 4 presents a simplified schematic model of health-system sustainability anchored on three interrelated pillars: research, policy, and delivery. Placing health promotion and disease prevention at the core of this framework is both pragmatic and necessary. Table 4 translates this conceptual model into practical policies and research priorities for advancing sustainable joint health. A life-course prevention continuum—encompassing primary (preventing modifiable risk factors or first joint injury), secondary (mitigating risk factor progression or early structural change), and tertiary (limiting disability and deferring surgery) prevention—aligns directly with the research–policy–delivery domains, reinforcing their collective role in advancing sustainable joint health. Research produces evidence by characterizing disease trajectories, identifying high-risk phenotypes, validating surrogate markers, and testing preventive and disease-modifying interventions. Policy translates research into priorities, incentives, and regulations that promote equity and sustainability. Delivery integrates evidence-based care pathways into clinical practice, community programs, and digital-health platforms to ensure implementation at scale. These three pillars form a feedback loop: delivery generates real-world data that refines research questions, research informs adaptive policy, and policy enables iterative improvements in delivery mechanisms. Successfully navigating the arthroplasty odyssey therefore demands simultaneous advancement across research, policy, and delivery—moving beyond continuity toward systemic transcendence.

Reimagining joint health through the lens of prevention, sustainability, and equity is no longer optional—it is imperative. The convergence of the rising prevalence of OA, finite material resources, and climate imperatives demands a paradigm shift from reactive surgical care to proactive, system-level interventions. This transformation must also account for the biomedical risks of current implant materials: in vivo corrosion and wear of cobalt–chromium alloys can release ions and particulate debris, which have been associated with local cytotoxicity, hypersensitivity, and systemic toxicities such as nephrotoxicity and cardiotoxicity [177,178,179]. Specifically, systemic arthroprosthetic cobaltism is an increasingly recognized multisystem complication of chrome-cobalt prosthesis wear or corrosion that can occur at relatively low blood cobalt concentrations, further complicating the risk–benefit assessment for widespread implant use [178]. Beyond clinical concerns, the ethical dimensions of implant production are substantial, with multiple investigative reports documenting child labor and exploitative conditions in the extraction of CRMs. A troubling paradox thus emerges: the same metals that expose mining communities to toxic hazards and exploitative conditions also pose biocompatibility risks when implanted in patients. Williams et al. [180] highlight the public health ramifications of cobalt mining in the Democratic Republic of the Congo, emphasizing the complex interdependence between global surgical demand and the adverse health and socioeconomic impacts experienced by local populations. This paradox underscores the urgency of reducing reliance on such high-risk materials, not only through technological innovation in implant design but also by addressing the root drivers of surgical demand. Prevention, rehabilitation, and lifestyle modification are practical strategies to delay or avoid surgery, thereby lowering dependence on implants that carry both biomedical and ethical costs. Such investments not only reduce the burden of disease and enhance QoL but also align with broader environmental and economic sustainability goals. As emphasized by the WHO’s One Health vision, the health of people, resilience of health systems, and integrity of our planet form a single, interconnected continuum. Our capacity to act decisively today will define not only the future of arthroplasty but also the ethical and ecological legacies we leave behind.

## 5. Concluding Remarks and Recommendations

In sum, the future of arthroplasty is contingent on the decisions we make today, offering a pivotal opportunity for transformative change. The direction in which orthopaedic surgery will evolve—whether it continues along a trajectory of incremental advancements or embarks on innovative pathways of preventive medicine—remains to be determined. As we embark on this journey, our shared objective is unequivocal: to improve the lives of individuals with OA in a way that guarantees responsible and sustainable use of healthcare resources for future generations [31]. The odyssey of arthroplasty, tracing from its inception to the possibilities that extend beyond 2050, is a tale of an unyielding commitment to excellence and innovation in the pursuit of enhancing human health. Encouragingly, this odyssey, although replete with challenges, presents a distinct chance to reimagine the future of joint healthcare, aspiring for the golden age of arthroplasty to evolve into an era marked by sustainability, inclusiveness, and patient-centered care [126].

At this critical juncture, the choices we make will determine whether arthroplasty practice follows a path of continuity or transcendence. By choosing transcendence, the community commits to a future in which the narrative of joint health is progressively rewritten with prevention at its core [144]. As Confucius simply stated, “A journey of a thousand miles begins with a single step”. This Perspective aims to be a first step—an opportunity to stimulate awareness and debate about recalibrating a largely reactive paradigm toward a genuinely proactive model that prioritizes prevention, equity, and system resilience and ultimately eradicates OA as a source of disability [2]. Such a shift will require courage, collaboration, and innovation, yet it also opens a promising horizon in which OA is no longer an inevitable fate but a preventable and manageable condition.

This alternative scenario anticipates a transformative future in which the paradigm decisively shifts from the prevalent REPLACE model of mass prosthesis deployment [46] to one that positions patients as empowered, proactive agents in their own care, inaugurating the early dawn of a prosumer-driven preventive era [118,120,128,130]. In this context, the prosumer—a synthesis of “producer” and “consumer”—embodies the theoretical shift from passive healthcare consumption toward *value co-creation*, wherein individuals actively participate in shaping, implementing, and sustaining their health outcomes [130]. Rooted in the concepts of participatory medicine and health democratization [128], the prosumer framework repositions patients as integral collaborators within the health ecosystem, generating shared responsibility and agency in disease prevention and management. Such a future challenges the orthopaedic field, together with rheumatology, to re-evaluate how joint health is managed, placing proactive OA prevention and timely interventions at the center of practice.

Adopting a prosumer-driven model involves equipping individuals with the knowledge, tools, and motivation to engage in preventive measures—which, however, requires sustained lifestyle modification, regular physical activity, and early diagnostic engagement—thereby prioritizing long-term joint health and QoL over default surgical solutions [52,123]. In this vision, patients become active co-contributors to their health outcomes, rather than passive recipients of care [118]. Nevertheless, translating a prosumer-driven approach into practice demands a coordinated package of policy levers, including financing and reimbursement mechanisms for preventive programs, integration of prevention into primary care through appropriate workforce training, scalable digital/community platforms for self-management education, and performance indicators/metrics oriented to equity and long-term joint health, implemented within the PREVENTive care framework (see Figure 3; Table 3) so that individual engagement yields demonstrable population-level outcomes.

Achieving this vision will require more than just policy. As schematically illustrated in Figure 4, the adoption of a prosumer-driven approach demands integrated and cooperative action across the health spectrum—clinicians, researchers, policymakers, and patients must work in concert [11,118,120,126,128,130]. This transition toward preventive, prosumer-centered care requires a fundamental reorientation of health-economic policies and investment strategies: financing models must reward prevention and long-term function, workforce development must prioritize prevention and primary care integration, and R&D priorities must shift toward low-burden, scalable interventions (Table 4). Although challenging, this transformation is essential for addressing the complexities of future demand. Thus, the future trajectory of arthroplasty beyond 2050 represents not only a continuation of its historical evolution but also a critical decision point: at this crossroads, the field must choose between preserving the status quo or embracing a prevention-first strategy for joint health.

Practical readiness is essential in this regard. To meet the projected surge in joint replacement demand, hospitals and health systems must engage in proactive planning, including capacity forecasting, strategic stock management and procurement, and contingency arrangements for critical components of joint replacement surgery [105]. Without such planning, systems risk escalating delays, price spikes, or outright shortages that would jeopardize timely access to life-changing surgery. International policy and guidance increasingly emphasize supply chain resilience and equitable allocation mechanisms [90,91,92], and the same principles should be applied to orthopaedic implants. In the absence of such measures, routine practice will be vulnerable to device delays and cost increases driven by raw material bottlenecks [36,84,85,86], with the greatest consequences falling on the most vulnerable patients and health systems (see Table 2 for details).

Accordingly, the response must be multifaceted: scale up preventive and conservative care programs that safely delay or avert surgery where appropriate; invest in registries and post-market surveillance that capture device performance, activity profiles, and equity metrics; incentivize procurement and research and development (R&D) approaches that favor low-material intensity, substitution, circular-economy solutions, and principles of an in-spiral economy [36,41,97], emphasizing the cyclical reuse and regeneration of resources to minimize waste and environmental impact; and embed equity and cultural representativeness in device testing and clinical guidance. These actions are mutually reinforcing: prevention reduces demand, resilient procurement cushions supply shocks, and robust surveillance safeguards the safety, efficacy, and equity of innovation.

In closing, this is a moment for decisive and coordinated action. The golden age of arthroplasty need not be the last triumph of unsustainable practice; with foresight and sensible policy, it can mature into an era defined by sustainability, fairness, and genuine improvements in population health. Let us move forward, knowing that the collective actions taken today will define the legacy of arthroplasty for generations to come.


**Key Policy Takeaways**

■Embed CRM management and supply chain resilience into health policy planning—align health procurement and capacity planning with net-zero transition scenarios and national resilience strategies to anticipate and mitigate shortages that could threaten arthroplasty delivery.■Reform procurement to reduce cost and material intensity—implement coordinated regional purchasing, equity-adjusted pricing, and procurement incentives that reward lower-material-intensity and sustainable implant designs.■Invest in registries and mandatory post-market surveillance—require registry enrolment and capture device performance, patient activity profiles (including culturally specific ADLs), and equity metrics to support lifecycle assessment, conditional approvals, and evidence-based procurement.■Scale prevention and conservative care—nationally fund and reimburse structured education, supervised exercise programs, and community self-management, and link these programs to registries to measure outcomes and surgical deferral.■Incentivize R&D and circularity pilots—prioritize funding and procurement preferences for substitution, recycling, and design-for-circularity, and support safe metal recovery and reuse pilots under strict safety and regulatory safeguards; ensure policies are sensitive to LMIC affordability and implementation constraints.■Commission independent scenario modelling and adopt interdisciplinary policy frameworks—support iterative, independent modelling of material-supply risks and mitigation options (substitution, recycling, demand reduction) and embed interdisciplinary approaches that bring together healthcare, environmental science, and supply chain management to guide medium-term policy-making.


## 6. Limitations

Despite the strengths of this study in providing a benchmark for the impact of the scarcity of CRMs on surgical management of OA within a fluctuating managed care context, its exploration of the future of arthroplasty services amid shifting healthcare, environmental, and material scarcity paradigms has several limitations, particularly those inherent in prospective data. Primarily, prosthetic demand projections derived from U.S. Medicare data [15] highlight the need for broader demographic validation, particularly targeting geographically diverse and demographically significant regions, including the European Union, China, India, and rapidly developing nations across Africa. Given the potential for advancements in medical technology and changes in global health policies, predicting the course of knee/hip OA introduces a degree of uncertainty [11]. The variability in the prevalence of this condition across different populations, coupled with data scarcity in lower-income countries, poses significant challenges in constructing accurate global models [7]. Additionally, increasing criticism of the KL classification system (see Table 1 for details) for early OA detection underscores significant methodological challenges in current research practices [24].

Based on Hubbert bell-shaped depletion models, resource forecasts suggest that molybdenum and cobalt will reach their production peaks around 2030 and 2142, respectively [53]. When the Law of the Minimum is applied, indicating that the scarcest mineral restricts the total production as the limiting factor, it becomes evident that the manufacturing of joint prostheses—which relies on cobalt–chromium–molybdenum alloys—faces a significant constraint [181]. This bottleneck might be even more critical than initially believed, as this study’s scope is limited to the Hubbert peak of chromium, projected for 2107 [53], potentially overlooking broader resource challenges. Importantly, concerns regarding the scarcity of CRMs for orthopaedic implants, based on current reserve and consumption estimates, may not account for future shifts in the technological, political, and economic landscapes, altering material demands [35,37,41,53,83,85,86]. The changing dynamics of mineral reserves, influenced by new discoveries, consumption trends, and socioeconomic factors, highlight the variability in the availability of critical materials, emphasizing the need for caution when treating data as absolute indicators of scarcity. It is important to acknowledge that long-range projections are inherently uncertain and must be treated as provisional estimates. Therefore, assumptions should be revisited on a routine basis as new surveillance, registry data, and materials research become available, ensuring that policy and planning remain adaptive rather than static.

Nevertheless, the comparative scenarios and illustrative trajectories presented in this study are conditional and sensitive to several identifiable assumptions. Key sources of uncertainty include (i) resource and reserve estimates and their revisions over time; (ii) by-product recovery and processing behavior; (iii) the pace and scale of clinical or industrial substitution (for example, the adoption of ceramic, PEEK/PEKK, or advanced polyethylene alternatives); (iv) the degree to which recycling and design-for-circularity are implemented at scale; and (v) abrupt policy, trade, or technological shocks. To make these dependencies concrete, an accelerated substitution pathway (widespread shift from Co–Cr–Mo alloys to ceramic or high-performance polymer implants) would be expected to lower the projected metal demand and attenuate material-stress trajectories; a rapid, industry-level rollout of effective implant recycling and design-for-recycling could shorten the projected supply shortfalls and dampen price volatility; conversely, trade restrictions or major geopolitical disruptions could generate abrupt, non-linear lead-time spikes that temporarily exacerbate access inequities. Appendix A lists the parameter sets and alternative assumption bundles used to illustrate these effects and presents sensitivity comparisons so that readers can judge how different plausible assumption sets change the comparative scenarios.

Another limitation arises from growing concerns over the ongoing decrease in diesel production and rising costs, which are critical for heavy machinery in the mining industry and could further exacerbate the scarcity of CRMs if these trends persist [53]. Given the current energy climate, “peak oil” not only intensifies existing challenges but also leads to scarcity of oil-derived polymers and plastics [32], which severely compromises the availability of polyethylene for joint implants—a significant limitation to the analysis presented in this study. Simultaneously, advances in additive manufacturing have accelerated the development of 3D-printed polymeric biomaterials, including biomimetic, bioresponsive, and bioactive scaffolds, which show promise for tissue engineering and orthopaedic applications [50,98,99]. Such advancements hold the potential to significantly reduce the dependency on metallic materials in the manufacturing of knee and hip prosthetics. Although the recycling and postmortem retrieval of orthopaedic implant alloys used in TJR face challenges owing to their thermodynamic complexity [41,53,83,97], which may be mitigated through future design and material innovations, anticipated demographic shifts suggest a potential decline in the global population during the 21st century [182], further complicating long-term demand projections. Moreover, the accessibility and affordability of TKA and THA are markedly influenced by the economic status of patients and the scope of their private insurance coverage [10,11,12]. Such variability contributes to substantial cost differences in these surgical procedures, which are closely related to the wide range of healthcare and social security systems worldwide.

In addition to these complexities, the call for a prosumer-driven, prevention-centric paradigm that emphasizes proactive measures and patient empowerment may overlook the challenges of implementing such strategies across diverse healthcare systems with varying levels of resources, workforce capacity, infrastructure, digital access, and cultural healthcare perceptions/attitudes toward prevention, as well as educational disparities in the disproportionate burden of disability in lower-socioeconomic communities [102,103,104,119,126,128,130,133,144,147,152]. Without explicit attention to equity, there is a real risk that a prosumer model could widen disparities, benefiting well-resourced populations while leaving vulnerable groups behind. Many public health systems—including the U.S.—lack the infrastructure and reimbursement models necessary to scale these interventions meaningfully. This gap represents a missed opportunity to reduce future surgical demand and enhance the QoL of OA patients at a considerably lower cost.

Beyond the disparities observed in high-income countries, an additional limitation of this analysis is the insufficient integration of evidence from LMICs. Although the global burden of OA is substantial [7,14,17,56,59,60,61,62,63,183], procedural volumes in these regions remain extremely low, reflecting structural barriers such as limited surgical capacity, out-of-pocket payment requirements, high implant costs, and fragile supply procurement systems. Recent studies demonstrate that safe TJR is feasible in LMIC tertiary centers when infrastructure and training are available; however, access is highly uneven, waiting lists are long, and outcome data are fragmented by the absence of comprehensive national registries [63,184,185]. These inequities imply that material scarcity and supply chain shocks may have disproportionately severe impacts in LMICs, where substitution options, recycling infrastructure, and diversified suppliers are more constrained than in high-income settings. Furthermore, preventive frameworks that emphasize early detection and lifestyle modifications must be tailored to health systems with limited resources, variable insurance coverage, and competing public health priorities. Therefore, incorporating a global South perspective is essential to ensure that projections of future demand and scarcity are not overgeneralized from U.S. or European data alone but instead reflect the heterogeneous realities of a worldwide epidemic.

Considering these limitations, notably from predictions using non-linear models that provide static views yet crucially highlight significant exponential trends, the next logical and critical step is to emphasize the importance of continuous research, engage in multidisciplinary dialogue [131], and develop adaptive self-management strategies for joint health in the context of CRM scarcity, which potentially affects joint replacement surgeries. Drawing on lessons from the COVID-19 pandemic, which emphasized the dual challenge of exponential public health crises and resource scarcity [21,42,89,90,91,92], this call for action underscores the urgent need for innovative and comprehensive approaches to address the imminent arthroplasty challenges in an era of rapid green industrialization and finite resources, thereby advocating for our responses to be meticulously guided by the principle of prudence.

## Figures and Tables

**Table 1:** KL system for classification of OA. Adapted from [24].

Grade	Description	Features
0	No OA	No osteophytes, normal joint space, no signs of sclerosis or bone deformity
1	Doubtful OA	Possible osteophytic lipping, joint space is normal or slightly decreased. No definite joint deformity or sclerosis
2	Mild OA	Definite osteophytes, appreciable joint space narrowing, no significant bone deformities
3	Moderate OA	Prominent osteophyte formation, marked joint-space narrowing, potential subchondral sclerosis (increased bone density), and subtle bony deformities
4	Severe OA	Large osteophytes, significant joint space narrowing, subchondral sclerosis, deformity of bones/joints, and cyst formation

**Table 2:** Theoretical phases of arthroplasty sustainability challenges across clinical, financial, and material domains, summarizing potential system-level stages under continued demand growth and material-supply stress, with indicative approximate horizons and primary systemic risks to inform policy and planning. The phase descriptors, operational indicators, and illustrative thresholds are conceptual and scenario-based, intended to support hypothesis generation and prospective monitoring, rather than serve as predictive or prescriptive estimates. Sources: [7,15,18,19,20,21,22,50,53,58,83,94].

Phase	Main Features	Indicative Timeframe and Uncertainty	Primary System-Level Risks
Early warning (critical stage)	Rapid exponential rise in primary TKA/THA volumes; rising peri-operative and rehabilitation demand; emerging short lead-time pressures on implant supply; localized capacity strain in operating rooms and recovery services	Short–medium term. Moderate uncertainty (driven by registry and administrative data trends)	Growing waitlists; regional access inequities; perioperative bottlenecks; localized procurement volatility; early workforce overload
System strain (financial non-viability)	Budget plateaus and diminishing marginal gains from efficiency; widening payer funding gaps; sustained supply-chain disruption; longer implant lead-times and routine backorders	Medium term. Higher uncertainty (sensitive to policy, reimbursement, and market dynamics)	Deferred or rationed care; increased out-of-pocket expenditure; cost shifting between payers; erosion of equity and quality of care; accelerated workforce attrition
Critical shortage (material constraints)	Severe CRM scarcity or major, sustained price shocks; geopolitical supply instability, logistical challenges and export restrictions; substantial reduction in the range of available implant models, leading to routine case delays and cancellations	Medium–long term. High uncertainty (contingent on global supply and markets, substitution/recycling success, and technological change)	Widespread case cancellations/delays; severe backlog accumulation; compromised clinical outcomes; amplified global inequities; disproportionate impact on LMICs and vulnerable populations; emergency procurement pressures and ethical allocation dilemmas

**Table 3:** PREVENTive care framework for knee and hip OA prevention and management. This comprehensive framework tackles the OA crisis by integrating policy innovations to address the estimated high demand for surgical interventions, such as TKA and THA, through strategic, multilevel prevention approaches. It outlines seven key policy principles, ranging from patient-centered care to evidence-based interventions and community-oriented initiatives. Data sources from [3,5,69,113,118,119,120,126,127,128,130,136,137,138,143,144,145,146,147,148,149,150].

Policy Principles ^1^	Description	Expected Outcomes
Patient-centered	Implement personalized care that prioritizes patient preferences and needs, ensuring active involvement in decision-making, and tailored rehabilitation programs involving a multidisciplinary team to restore function, strength, and mobility in OA patients	Enhanced patient satisfaction and treatment adherence, improved recovery rates, reduced disability, and better overall QoL
Risk reducing	Develop and enforce strategies for injury prevention in sports, workplaces, and road safety. Collaborate with urban planners to design supportive built environments aimed at minimizing the risk of falls and fractures	Reduced incidence of joint injuries and PTOA, improved joint health, and decreased need for surgical interventions such as TKA and THA
Equity, inclusiveness, accessibility	Ensure equitable access to OA prevention and treatment programs across all socioeconomic, racial/ethnic, and geographic groups	Reduced disparities in OA prevalence and care outcomes, leading to improved QoL for marginalized populations
Vigilant	Establish comprehensive surveillance systems for monitoring OA and PTOA, utilizing advanced data analytics for timely intervention	Early detection of OA trends and risk factors, enabling proactive management, better disease control, and reduced long-term healthcare costs
Evidence-based	Formulate policies and treatment guidelines based on robust scientific evidence. Regularly assess the economic impact and cost-effectiveness of interventions	Improved patient outcomes, enhanced policy effectiveness, optimized use of healthcare resources, and reduced costs associated with OA management
Nurturing health promotion	Launch public health initiatives that promote joint health through education, community programs, and lifestyle interventions. Focus on non-pharmacological strategies	Increased public awareness and adoption of healthy behaviors, leading to early detection and prevention of OA, improved joint function, and overall well-being within urban and rural communities
Transparency	Ensure clear communication of treatment options, risks, and benefits to individuals. Improve transparency in healthcare data sharing and clinician-patient interactions	Increased patient trust and satisfaction, informed decision-making, and enhanced effectiveness of public health policies and clinical practices in OA management

^1^ The first letters of each policy principle form the acronym PREVENT, representing the foundational elements of the PREVENTive care framework.

**Table 4:** Prevention levels for OA mapped to objectives, typical timing/target populations, and recommended key outcome measures linked to the research, policy, and delivery pillars.

Level/Objective	Typical Timing/Target Population	Key Outcome Measures ^1^	Linked Pillar(s)
Primary prevention/Prevent development of risk factors or first joint injury	Childhood, adolescence, early adulthood; general population and at-risk groups	Incidence of index injuries; prevalence of obesity; biomechanical measures; adherence/process metrics	Research (efficacy), policy (population programs), delivery (community/school implementation)
Secondary prevention/Modify risk factors or halt progression from early structural change to symptomatic OA	Immediately post-injury or when early structural changes detected; at-risk cohorts	Change in risk factor (BMI, strength); imaging surrogates (MRI compositional measures, cartilage T2); symptom scores; time-to-symptomatic OA	Research (surrogates), delivery (clinical pathways), policy (coverage)
Tertiary prevention/Reduce progression to advanced disease, disability, or surgery	Symptomatic OA with established disease; patients at risk of rapid progression	Time-to-joint replacement; pain/function PROMs; health-related QoL; healthcare utilization/costs	Delivery (care pathways), policy (access, financing), research (comparative effectiveness)

^1^ Outcome selection should match prevention intent (prevent risk factor vs. modify risk factor vs. prevent disease/illness); intermediate imaging or biomarker surrogates require explicit justification and evidence of linkage to clinically meaningful endpoints [177,178,179].

## Data Availability

No new data were created or analyzed in this study. Data sharing is not applicable to this article.

## Abbreviations

The following abbreviations are used in this manuscript:

ACL	Anterior cruciate ligament
ADLs	Activities of daily living
AI	Artificial intelligence
BAU	Business-as-usual
BMI	Body Mass Index
CMS	Centers for Medicare & Medicaid Services
CPRD	Clinical Practice Research Datalink
COVID-19	Coronavirus disease 2019
CRMs	Critical raw materials
CT	Computed Tomography
HDPE	High-density polyethylene
IPCC	Intergovernmental Panel on Climate Change
JSN	Joint space narrowing
KL	Kellgren-Lawrence
LLDPE	Linear low-density polyethylene
LLMs	Large language models
LMICs	Low- and middle-income countries
MDPE	Medium-density polyethylene
MEP	Movement-evoked pain
MRI	Magnetic Resonance Imaging
NZE	Net Zero Emissions
OA	Osteoarthritis
OARSI	Osteoarthritis Research Society International
PREVENT	Patient-centered, Risk reducing, Equity, inclusiveness, accessibility, Vigilant, Evidence-based, Nurturing health promotion, Transparency
PEEK	Polyetheretherketone
PEKK	Polyetherketoneketone
PROMs	Patient-reported outcome measures
PRP	Platelet-rich plasma
PTOA	Post-traumatic OA
QoL	Quality of life
REPLACE	Reactive End-stage Prosthesis for Load-bearing Arthritic Cartilage Erosion
R&D	Research and development
THA	Total hip arthroplasty
TJR	Total joint replacement
TKA	Total knee arthroplasty
UHMWPE	Ultra-high-molecular-weight polyethylene
U.S.	United States
WHO	World Health Organization
XPE	Cross-linked polyethylene

## Appendix A

Table 2 presents illustrative comparative constructs rather than empirical or deterministic forecasts. Their purpose is to stimulate discussion and hypothesis generation regarding potential co-occurring clinical, financial, and material risks under business-as-usual (BAU) assumptions. The trajectories and thresholds are conditional and scenario-based, drawing on the parameter choices/ranges and uncertainty bounds discussed in the cited literature (refs. [15,53,174]). Accordingly, they are conceptual scaffolds designed to guide future empirical testing rather than provide definitive projections or policy thresholds. To promote interpretive clarity, the qualitative phases in Table 2 were aligned with explicit yet hypothetical operational indicators and illustrative triggers, serving as monitoring constructs and prospective test variables, rather than prescriptive benchmarks. In the clinical domain, indicative metrics included the annual percentage growth in primary TKA/THA volumes (three-year rolling average), mean waitlist duration (weeks), and perioperative throughput (procedures per operating theatre per week). These parameters reflect values typically reported in international registries and perioperative capacity studies (refs. [13,20,94]). A hypothetical early warning threshold might involve a sustained three-year procedure growth exceeding 5% combined with an average supply lead-time greater than 14 days for two or more consecutive years. In the financial domain, illustrative indicators include nominal orthopaedic budget growth relative to procedural demand, real-term payer reimbursement trends, and the out-of-pocket share of arthroplasty costs (refs. [149,186,187]). A representative system strain condition could be indicated when the real orthopaedic budget growth remains below 1% while the annual demand growth exceeds 3% for three or more consecutive years, or when the supply stockout frequency surpasses 5% within a 12-month period. In the material domain, central indicators comprise the mean supply lead-time, procurement stockout frequency, and annual price volatility (refs. [37,53,83]). A hypothetical critical shortage condition could be defined by a sustained decline in CRM production—approximated by the median outcome of a Hubbert-style depletion curve— resulting in a projected supply shortfall greater than X% relative to baseline demand for more than Y years, or by annual price volatility exceeding Z% for at least two consecutive years. These operational mappings are provided as a conceptual roadmap—not as validated or fixed decision thresholds—for ongoing surveillance and empirical testing of systemic resilience. They are intended to facilitate transparent, cross-disciplinary dialogue among clinical, economic, and materials science communities and to provide a shared language for future monitoring and research on the long-term sustainability of arthroplasty services.

## References

[1] Briggs A.M., Cross M.J., Hoy D.G., Sánchez-Riera L., Blyth F.M., Woolf A.D., March L. (2016). Musculoskeletal health conditions represent a global threat to healthy aging: A report for the 2015 World Health Organization World Report on Ageing and Health. Gerontologist.

[2] GBD 2021 Diseases and Injuries Collaborators (2024). Global incidence, prevalence, years lived with disability (YLDs), disability-adjusted life-years (DALYs), and healthy life expectancy (HALE) for 371 diseases and injuries in 204 countries and territories and 811 subnational locations, 1990-2021: A systematic analysis for the Global Burden of Disease Study 2021. Lancet.

[3] Jennifer M.H., Charles G.H., Teresa J.B. (2012). A public health approach to addressing arthritis in older adults: The most common cause of disability. Am. J. Public Health.

[4] Maradit Kremers H., Larson D.R., Crowson C.S., Kremers W.K., Washington R.E., Steiner C.A., Jiranek W.A., Berry D.J. (2015). Prevalence of total hip and knee replacement in the United States. J. Bone Jt. Surg. Am..

[5] Deshpande B.R., Katz J.N., Solomon D.H., Yelin E.H., Hunter D.J., Messier S.P., Suter L.G., Losina E. (2016). Number of persons with symptomatic knee osteoarthritis in the US: Impact of race and ethnicity, age, sex, and obesity. Arthritis Care Res..

[6] Hunter D.J., March L., Chew M. (2020). Osteoarthritis in 2020 and beyond: A Lancet Commission. Lancet.

[7] Steinmetz J.D., Culbreth G.T., Haile L.M., Rafferty Q., Lo J., Fukutaki K.G., Cruz J.A., Smith A.E., Vollset S.E., Brooks P.M. (2023). Global, regional, and national burden of osteoarthritis, 1990-2020 and projections to 2050: A systematic analysis for the Global Burden of Disease Study 2021. Lancet Rheumatol..

[8] Centers for Medicare and Medicaid Services Comprehensive Care for Joint Replacement Model. https://www.cms.gov/priorities/innovation/innovation-models/cjr.

[9] Learmonth I.D., Young C., Rorabeck C. (2007). The operation of the century: Total hip replacement. Lancet.

[10] Kurtz S.M., Ong K.L., Schmier J., Mowat F., Saleh K., Dybvik E., Karrholm J., Garellick G., Havelin L.I., Furnes O. (2007). Future clinical and economic impact of revision total hip and knee arthroplasty. J. Bone Jt. Surg. Am..

[11] Wilson N.A., Schneller E.S., Montgomery K., Bozic K.J. (2008). Hip and knee implants: Current trends and policy considerations. Health Aff..

[12] Bitton R. (2009). The economic burden of osteoarthritis. Am. J. Manag. Care.

[13] Kurtz S., Ong K., Lau E., Mowat F., Halpern M. (2007). Projections of primary and revision hip and knee arthroplasty in the United States from 2005 to 2030. J. Bone Jt. Surg. Am..

[14] Culliford D., Maskell J., Judge A., Cooper C., Prieto-Alhambra D., Arden N.K. (2015). Future projections of total hip and knee arthroplasty in the UK: Results from the UK Clinical Practice Research Datalink. Osteoarthr. Cartil..

[15] Shichman I., Roof M., Askew N., Nherera L., Rozell J.C., Seyler T.M., Schwarzkopf R. (2023). Projections and epidemiology of primary hip and knee arthroplasty in medicare patients to 2040-2060. JB JS Open Access.

[16] Singh J.A., Yu S., Chen L., Cleveland J.D. (2019). Rates of total joint replacement in the United States: Future projections to 2020–2040 using the national inpatient sample. J. Rheumatol..

[17] Ackerman I.N., Bohensky M.A., Zomer E., Tacey M., Gorelik A., Brand C.A., de Steiger R. (2019). The projected burden of primary total knee and hip replacement for osteoarthritis in Australia to the year 2030. BMC Musculoskelet. Disord..

[18] Clement N.D., Scott C.E.H., Murray J.R.D., Howie C.R., Deehan D.J. (2021). The number of patients “worse than death” while waiting for a hip or knee arthroplasty has nearly doubled during the COVID-19 pandemic. Bone Jt. J..

[19] Farrow L., Gardner W.T., Tang C.C., Low R., Forget P., Ashcroft G.P. (2023). Impact of COVID-19 on opioid use in those awaiting hip and knee arthroplasty: A retrospective cohort study. BMJ Qual. Saf..

[20] Jabbal M., Burt J., Clarke J., Moran M., Walmsley P., Jenkins P.J. (2023). Trends in incidence and average waiting time for arthroplasty from 1998-2021: An observational study of 282,367 patients from the Scottish arthroplasty project. Ann. R. Coll. Surg. Engl..

[21] Sniderman J., Abdeen A. (2023). The impact of the COVID-19 pandemic on the practice of hip and knee arthroplasty. JBJS Rev..

[22] French J.M.R., Deere K., Jones T., Pegg D.J., Reed M.R., Whitehouse M.R., Sayers A. (2024). An analysis of the effect of the COVID-19-induced joint replacement deficit in England, Wales, and Northern Ireland suggests recovery will be protracted. Bone Jt. J..

[23] Huynh C., Puyraimond-Zemmour D., Maillefert J.F., Conaghan P.G., Davis A.M., Gunther K.P., Hawker G., Hochberg M.C., Kloppenburg M., Lim K. (2018). Factors associated with the orthopaedic surgeon’s decision to recommend total joint replacement in hip and knee osteoarthritis: An international cross-sectional study of 1905 patients. Osteoarthr. Cartil..

[24] Kohn M.D., Sassoon A.A., Fernando N.D. (2016). Classifications in brief: Kellgren-Lawrence classification of osteoarthritis. Clin. Orthop. Relat. Res..

[25] Orchard J.W., Tutt L.E., Hines A., Orchard J.J. (2025). Associations between common hip and knee osteoarthritis treatments and all-cause mortality. Healthcare.

[26] Lee K., Goodman S.B. (2008). Current state and future of joint replacements in the hip and knee. Expert Rev. Med. Devices.

[27] Lo Y.C., Chen Y.P., Lin H.E., Chang W.C., Ho W.P., Jang J.P., Kuo Y.J. (2025). Factors associated with decisional regret after shared decision making for patients undergoing total knee arthroplasty. Healthcare.

[28] Gunaratne R., Pratt D.N., Banda J., Fick D.P., Khan R.J.K., Robertson B.W. (2017). Patient dissatisfaction following total knee arthroplasty: A systematic review of the literature. J. Arthroplast..

[29] Bourne R.B., Chesworth B.M., Davis A.M., Mahomed N.N., Charron K.D. (2010). Patient satisfaction after total knee arthroplasty: Who is satisfied and who is not?. Clin. Orthop. Relat. Res..

[30] Cronström A., Dahlberg L.E., Nero H., Hammarlund C.S. (2019). “I was considering surgery because I believed that was how it was treated”: A qualitative study on willingness for joint surgery after completion of a digital management program for osteoarthritis. Osteoarthr. Cartil..

[31] United Nations (2023). The Sustainable Development Goals Report 2023.

[32] IEA (2021). Net Zero by 2050.

[33] Delaie C., Cerlier A., Argenson J.N., Escudier J.C., Khakha R., Flecher X., Jacquet C., Ollivier M. (2023). Ecological burden of modern surgery: An analysis of total knee replacement’s life cycle. Arthroplast. Today.

[34] IPCC, 2018: Summary for Policymakers.

[35] Eggert R.G. (2011). Minerals go critical. Nat. Chem..

[36] Raabe D. (2023). The materials science behind sustainable metals and alloys. Chem. Rev..

[37] USGS (2025). Mineral Commodity Summaries 2025.

[38] Moradlou H., Reefke H., Skipworth H., Roscoe S. (2021). Geopolitical disruptions and the manufacturing location decision in multinational company supply chains: A Delphi study on Brexit. Int. J. Oper. Prod. Manag..

[39] Gulley A.L. (2023). China, the Democratic Republic of the Congo, and artisanal cobalt mining from 2000 through 2020. Proc. Natl. Acad. Sci. USA.

[40] Nze-Ekpebie R.A. (2023). Global supply chain effects on medical devices. J. Healthc. Commun..

[41] Watari T., Nansai K., Nakajima K. (2020). Review of critical metal dynamics to 2050 for 48 elements. Resour. Conserv. Recycl..

[42] Bhaskar S., Tan J., Bogers M., Minssen T., Badaruddin H., Israeli-Korn S., Chesbrough H. (2020). At the epicenter of COVID-19—the tragic failure of the global supply chain for medical supplies. Front. Public Health.

[43] (2021). National Strategy for a Resilient Public Health Supply Chain.

[44] International Energy Agency (2022). The Role of Critical Minerals in Clean Energy Transitions.

[45] Energy Transitions Commission (2023). Material and Resource Requirements for the Energy Transition.

[46] Hudak P.L., Clark S.J., Raymond G. (2013). The omni-relevance of surgery: How medical specialization shapes orthopedic surgeons’ treatment recommendations. Health Commun..

[47] Quintana J.M., Escobar A., Arostegui I., Bilbao A., Azkarate J., Goenaga J.I., Arenaza J.C. (2006). Health-related quality of life and appropriateness of knee or hip joint replacement. JAMA Intern. Med..

[48] Konttinen Y.T., Milosev I., Trebse R., Rantanen P., Linden R., Tiainen V.M., Virtanen S., Revell P.A. (2008). 6—Metals for joint replacement. Joint Replacement Technology.

[49] Merola M., Affatato S. (2019). Materials for hip prostheses: A review of wear and loading considerations. Materials.

[50] Szczęsny G., Kopec M., Politis D.J., Kowalewski Z.L., Łazarski A., Szolc T. (2022). A review on biomaterials for orthopaedic surgery and traumatology: From past to present. Materials.

[51] Ratti M., Ceriotti D., Rescinito R., Bibi R., Panella M. (2024). Does robotic assisted technique improve patient utility in total knee arthroplasty? A comparative retrospective cohort study. Healthcare.

[52] Hunter D.J., Bierma-Zeinstra S. (2019). Osteoarthritis. Lancet.

[53] Calvo G., Valero A., Valero A. (2017). Assessing maximum production peak and resource availability of non-fuel mineral resources: Analyzing the influence of extractable global resources. Resour. Conserv. Recycl..

[54] OECD (2014). Geographic Variations in Health Care: What Do We Know and What Can Be Done to Improve Health System Performance?.

[55] Jennison T., MacGregor A., Goldberg A. (2023). Hip arthroplasty practice across the Organisation for Economic Co-operation and Development (OECD) over the last decade. Ann. R. Coll. Surg. Engl..

[56] Lübbeke A., Silman A.J., Barea C., Prieto-Alhambra D., Carr A.J. (2018). Mapping existing hip and knee replacement registries in Europe. Health Policy.

[57] Rupp M., Lau E., Kurtz S.M., Alt V. (2020). Projections of primary TKA and THA in Germany from 2016 through 2040. Clin. Orthop. Relat. Res..

[58] Matharu G.S., Culliford D.J., Blom A.W., Judge A. (2022). Projections for primary hip and knee replacement surgery up to the year 2060: An analysis based on data from The National Joint Registry for England, Wales, Northern Ireland and the Isle of Man. Ann. R. Coll. Surg. Engl..

[59] Cao X., Zhu R., Liu D., Cheng Y., Sun Y., Huang Z. (2025). Epidemiological trends in burden of osteoarthritis in China: An analysis from 1990 to 2021 with forecasts for 2022-2050. Front. Public Health.

[60] Feng B., Zhu W., Bian Y.Y., Chang X., Cheng K.Y., Weng X.S. (2020). China artificial joint annual data report. Chin. Med. J..

[61] Vaidya S.V., Jogani A.D., Pachore J.A., Armstrong R., Vaidya C.S. (2020). India joining the world of hip and knee registries: Present status—a leap forward. Indian J. Orthop..

[62] Davies P.S., Graham S.M., Maqungo S., Harrison W.J. (2019). Total joint replacement in sub-Saharan Africa: A systematic review. Trop. Doct..

[63] Laubscher K., Dey R., Nortje M., Held M., Kauta N. (2022). Primary hip and knee arthroplasty at district level is safe and may reduce the burden on tertiary care in a low-income setting. BMC Musculoskelet. Disord..

[64] Wang A.Y., Wong M.S., Humbyrd C.J. (2018). Eligibility criteria for lower extremity joint replacement may worsen racial and socioeconomic disparities. Clin. Orthop. Relat. Res..

[65] Thirukumaran C.P., Cai X., Glance L.G., Kim Y., Ricciardi B.F., Fiscella K.A., Li Y. (2020). Geographic variation and disparities in total joint replacement Use for Medicare beneficiaries: 2009 to 2017. J. Bone Jt. Surg. Am..

[66] Thirukumaran C.P., Kim Y., Cai X., Ricciardi B.F., Li Y., Fiscella K.A., Mesfin A., Glance L.G. (2021). Association of the comprehensive care for joint replacement model with disparities in the use of total hip and total knee replacement. JAMA Netw. Open.

[67] Goodman S.M., Mannstadt I., Gibbons J.A.B., Rajan M., Bass A., Russell L., Mehta B., Figgie M., Parks M.L., Venkatachalam S. (2023). Healthcare disparities: Patients’ perspectives on barriers to joint replacement. BMC Musculoskelet Disord..

[68] Amen T.B., Liimakka A.P., Jain B., Rudisill S.S., Bedair H.S., Chen A.F. (2023). Total joint arthroplasty utilization after orthopaedic surgery referral: Identifying disparities along the care pathway. J. Arthroplast..

[69] Malchau H., Bragdon C.R., Muratoglu O.K. (2011). The stepwise introduction of innovation into orthopedic surgery: The next level of dilemmas. J. Arthroplast..

[70] Inabathula A., Semerdzhiev D.I., Srinivasan A., Amirouche F., Puri L., Piponov H. (2024). Robots on the stage: A snapshot of the American robotic total knee arthroplasty market. JB JS Open Access.

[71] Wu H., Yao S., Bao H., Guo Y., Xu C., Ma J. (2025). ChatGPT-4.0 and DeepSeek-R1 does not yet provide clinically supported answers for knee osteoarthritis. Knee.

[72] Barakat N., Ramamurti P., Duensing I.M., Browne J.A. (2024). Financial conflicts of interest and industry funding are associated with conclusions favorable to new technologies: A review of published economic analyses in hip and knee arthroplasty. J. Arthroplast..

[73] Peters R.M., Ten Have B., Rykov K., Van Steenbergen L., Putter H., Rutgers M., Vos S., Van Steijnen B., Poolman R.W., Vehmeijer S.B.W. (2022). The learning curve of the direct anterior approach is 100 cases: An analysis based on 15,875 total hip arthroplasties in the Dutch Arthroplasty Register. Acta Orthop..

[74] Sarpong N.O., Herndon C.L., Held M.B., Neuwirth A.L., Hickernell T.R., Geller J.A., Cooper H.J., Shah R.P. (2020). What is the learning curve for new technologies in total joint arthroplasty? A review. Curr. Rev. Musculoskelet. Med..

[75] Ravi B., Jenkinson R., Austin P.C., Croxford R., Wasserstein D., Escott B., Paterson J.M., Kreder H., Hawker G.A. (2014). Relation between surgeon volume and risk of complications after total hip arthroplasty: Propensity score matched cohort study. BMJ.

[76] Patel R.V., Gonzalez M.R., Attaar N., Patel M.V., Lozano-Calderon S.A. (2025). Analyzing orthopaedic workforce trends in an ever-changing landscape. J. Am. Acad. Orthop. Surg..

[77] Rullán P.J., Deren M.E., Zhou G., Emara A.K., Klika A.K., Schiltz N.K., Barsoum W.K., Koroukian S., Piuzzi N.S. (2023). The arthroplasty surgeon growth indicator: A tool for monitoring supply and demand trends in the orthopaedic surgeon workforce from 2020 to 2050. J. Bone Jt. Surg. Am..

[78] Cheng H.Y., Beswick A.D., Bertram W., Siddiqui M.A., Gooberman-Hill R., Whitehouse M.R., Wylde V. (2025). What proportion of people have long-term pain after total hip or knee replacement? An update of a systematic review and meta-analysis. BMJ Open.

[79] Ayers D.C., Yousef M., Zheng H., Yang W., Franklin P.D. (2022). The prevalence and predictors of patient dissatisfaction 5-years following primary total knee arthroplasty. J. Arthroplast..

[80] Ames S.E., Cowan J.B., Kenter K., Emery S., Halsey D. (2017). Burnout in orthopaedic surgeons: A challenge for leaders, learners, and colleagues: AOA critical issues. J. Bone Jt. Surg. Am..

[81] Mihcin S., Sahin A.M., Yilmaz M., Alpkaya A.T., Tuna M., Akdeniz S., Korkmaz N.C., Tosun A., Sahin S. (2023). Database covering the prayer movements which were not available previously. Sci. Data.

[82] Sorenson C., Drummond M. (2014). Improving medical device regulation: The United States and Europe in perspective. Milbank. Q..

[83] Graedel T.E., Harper E.M., Nassar N.T., Reck B.K. (2015). On the materials basis of modern society. Proc. Natl. Acad. Sci. USA.

[84] Valero A., Valero A., Calvo G., Ortego A. (2018). Material bottlenecks in the future development of green technologies. Renew. Sustain. Energy Rev..

[85] Rhodes C.J. (2019). Endangered elements, critical raw materials and conflict minerals. Sci. Prog..

[86] Girtan M., Wittenberg A., Grilli M.L., de Oliveira D.P.S., Giosue C., Ruello M.L. (2021). The critical raw materials issue between scarcity, supply risk, and unique properties. Materials.

[87] Murphy L.B., Cisternas M.G., Theis K.A., Brady T.J., Bohm M.K., Guglielmo D., Hootman J.M., Barbour K.E., Boring M.A., Helmick C.G. (2020). All-cause opioid prescriptions dispensed: The outsized role of adults with arthritis. Am. J. Prev. Med..

[88] Dowell D., Ragan K.R., Jones C.M., Baldwin G.T., Chou R. (2022). CDC clinical practice guideline for prescribing opioids for pain — United States. MMWR Recomm. Rep..

[89] Sharma A., Gupta P., Jha R. (2020). COVID-19: Impact on health supply chain and lessons to be learnt. J. Health Man..

[90] Emanuel E.J., Persad G. (2023). The shared ethical framework to allocate scarce medical resources: A lesson from COVID-19. Lancet.

[91] OECD (2023). Ready for the Next Crisis? Investing in Health System Resilience.

[92] (2024). OECD. Securing Medical Supply Chains in a Post-Pandemic World.

[93] OECD (2022). The Supply of Critical Raw Materials Endangered by Russia’s War on Ukraine.

[94] Hurst J., Siciliani L. (2003). Tackling Excessive Waiting Times for Elective Surgery: A Comparison of Policies in Twelve OECD Countries.

[95] Porter G.M., Balian J., Ng A.P., Mannings H., Jeffcoat D.M., Benharash P. (2025). Cost-volume analysis of primary total knee and hip arthroplasty in the United States. J. Arthroplast..

[96] Hannon C.P., Goodman S.M., Austin M.S., Yates A., Guyatt G., Aggarwal V.K., Baker J.F., Bass P., Bekele D.I., Dass D. (2023). 2023 American College of Rheumatology and American Association of Hip and Knee Surgeons Clinical Practice Guideline for the optimal timing of elective hip or knee arthroplasty for patients with symptomatic moderate-to-severe osteoarthritis or advanced symptomatic osteonecrosis with secondary arthritis for whom nonoperative therapy is ineffective. Arthritis Care Res..

[97] Valero A., Valero A. (2019). Thermodynamic rarity and recyclability of raw materials in the energy transition: The need for an in-spiral economy. Entropy.

[98] Sarfraz S., Mäntynen P.H., Laurila M., Rossi S., Leikola J., Kaakinen M., Suojanen J., Reunanen J. (2022). Comparison of titanium and PEEK medical plastic implant materials for their bacterial biofilm formation properties. Polymers.

[99] Al-Shalawi F.D., Mohamed Ariff A.H., Jung D.W., Mohd Ariffin M.K.A., Seng Kim C.L., Brabazon D., Al-Osaimi M.O. (2023). Biomaterials as implants in the orthopedic field for regenerative medicine: Metal versus synthetic polymers. Polymers.

[100] Said A.I., Patricia D.F. (2013). Race and elective joint replacement: Where a disparity meets patient preference. Am. J. Public Health.

[101] Arcaya M.C., Figueroa J.F. (2017). Emerging trends could exacerbate health inequities in the United States. Health Aff..

[102] Cogburn C.D. (2019). Culture, race, and health: Implications for racial inequities and population health. Milbank. Q..

[103] Faison W.E., Harrell P.G., Semel D. (2021). Disparities across diverse populations in the health and treatment of patients with osteoarthritis. Healthcare.

[104] Cullen M.R., Lemeshow A.R., Russo L.J., Barnes D.M., Ababio Y., Habtezion A. (2022). Disease-specific health disparities: A targeted review focusing on race and ethnicity. Healthcare.

[105] Humphreys P., Spratt B., Tariverdi M., Burdett R.L., Cook D., Yarlagadda P., Corry P. (2022). An overview of hospital capacity planning and optimisation. Healthcare.

[106] Callahan L.F., Ambrose K.R., Albright A.L., Altpeter M., Golightly Y.M., Huffman K.F., Nelson A.E., Weisner S.E. (2019). Public Health Interventions for Osteoarthritis - updates on the Osteoarthritis Action Alliance’s efforts to address the 2010 OA Public Health Agenda Recommendations. Clin. Exp. Rheumatol..

[107] Jinks C., Botto-van Bemden A., Bunzli S., Bowden J., Egerton T., Eyles J., Foster N., Healey E.L., Maddison J., O’Brien D. (2024). Changing the narrative on osteoarthritis: A call for global action. Osteoarthr. Cartil..

[108] Nguyen A., Lee P., Rodriguez E.K., Chahal K., Freedman B.R., Nazarian A. (2025). Addressing the growing burden of musculoskeletal diseases in the ageing US population: Challenges and innovations. Lancet Healthy Longev..

[109] Brandt K.D., Dieppe P., Radin E.L. (2008). Etiopathogenesis of osteoarthritis. Rheum. Dis. Clin. N. Am..

[110] Goldring M.B., Otero M. (2011). Inflammation in osteoarthritis. Curr. Opin. Rheumatol..

[111] Scanzello C.R., Goldring S.R. (2012). The role of synovitis in osteoarthritis pathogenesis. Bone.

[112] Houard X., Goldring M.B., Berenbaum F. (2013). Homeostatic mechanisms in articular cartilage and role of inflammation in osteoarthritis. Curr. Rheumatol. Rep..

[113] Chu C.R., Andriacchi T.P. (2015). Dance between biology, mechanics, and structure: A systems-based approach to developing osteoarthritis prevention strategies. J. Orthop. Res..

[114] Whittaker J.L., Runhaar J., Bierma-Zeinstra S., Roos E.M. (2021). A lifespan approach to osteoarthritis prevention. Osteoarthr. Cartil..

[115] Woolf A.D. (2000). The bone and joint decade 2000-2010. Ann. Rheum. Dis..

[116] Lidgren L. (2003). The bone and joint decade 2000-2010. Bull. World Health Organ..

[117] Osteoarthritis Research Society International (OARSI) (2016). Osteoarthritis: A Serious Disease.

[118] Young A., Flower L. (2002). Patients as partners, patients as problem-solvers. Health Commun..

[119] Foote S.B., Blewett L.A. (2003). Politics of prevention: Expanding prevention benefits in the Medicare program. J. Public Health Pol..

[120] Dumay A.C.M., Blank J.L.T., Bos L., Carroll D., Kun L., Marsh A., Roa L.M. (2010). Healthcare prosumerism. Future Visions on Biomedicine and Bioinformatics 1: A Liber Amicorum in Memory of Swamy Laxminarayan.

[121] de Melo L.R.S., Hunter D., Fortington L., Peeters A., Seward H., Vertullo C., Hills A.P., Brown W., Feng Y., Lloyd D.G. (2020). National Osteoarthritis Strategy brief report: Prevention of osteoarthritis. Aust. J. Gen. Pract..

[122] Ambrose K.R., Huffman K.F., Odom E.L., Foster A.L., Turkas N., Callahan L.F. (2024). A public health approach to osteoarthritis in the United States. Osteoarthr. Cartil..

[123] Berenbaum F., Wallace I.J., Lieberman D.E., Felson D.T. (2018). Modern-day environmental factors in the pathogenesis of osteoarthritis. Nat. Rev. Rheumatol..

[124] Noto S. (2023). Perspectives on aging and quality of life. Healthcare.

[125] del Río E. (2025). Thick or thin? Implications of cartilage architecture for osteoarthritis risk in sedentary lifestyles. Biomedicines.

[126] Bircher J., Kuruvilla S. (2014). Defining health by addressing individual, social, and environmental determinants: New opportunities for health care and public health. J. Public Health Pol..

[127] Ali A., Katz D.L. (2015). Disease prevention and health promotion: How integrative medicine fits. Am. J. Prev. Med..

[128] Batalden M., Batalden P., Margolis P., Seid M., Armstrong G., Opipari-Arrigan L., Hartung H. (2016). Coproduction of healthcare service. BMJ Qual. Saf..

[129] Chauvin J., Perera Y., Clarke M. (2016). Digital technologies for population health and health equity gains: The perspective of public health associations. J. Public Health Pol..

[130] Russo G., Moretta Tartaglione A., Cavacece Y. (2018). Empowering patients to co-create a sustainable healthcare value. Sustainability.

[131] Andriacchi T.P., Griffin T.M., Loeser R.F., Chu C.R., Roos E.M., Hawker G.A., Erhart-Hledik J.C., Fischer A.G. (2020). Bridging Disciplines as a pathway to Finding New Solutions for Osteoarthritis a collaborative program presented at the 2019 Orthopaedic Research Society and the Osteoarthritis Research Society International. Osteoarthr. Cartil. Open.

[132] Goulbourne T., Yanovitzky I. (2021). The communication infrastructure as a social determinant of health: Implications for health policymaking and practice. Milbank. Q..

[133] González-Cacheda B., Outeda C.C. (2025). Understanding attitudes, knowledge, and use of e-health services in the health system in Spain. J. Public Health Pol..

[134] Gibbs A.J., Gray B., Wallis J.A., Taylor N.F., Kemp J.L., Hunter D.J., Barton C.J. (2023). Recommendations for the management of hip and knee osteoarthritis: A systematic review of clinical practice guidelines. Osteoarthr. Cartil..

[135] Kolasinski S.L., Neogi T., Hochberg M.C., Oatis C., Guyatt G., Block J., Callahan L., Copenhaver C., Dodge C., Felson D. (2020). 2019 American College of Rheumatology/Arthritis Foundation Guideline for the management of osteoarthritis of the hand, hip, and knee. Arthritis Care Res..

[136] Kramer W.C., Hendricks K.J., Wang J. (2011). Pathogenetic mechanisms of posttraumatic osteoarthritis: Opportunities for early intervention. Int. J. Clin. Exp. Med..

[137] Zhang W., Moskowitz R.W., Nuki G., Abramson S., Altman R.D., Arden N., Bierma-Zeinstra S., Brandt K.D., Croft P., Doherty M. (2008). OARSI recommendations for the management of hip and knee osteoarthritis. Part II: OARSI evidence-based, expert consensus guidelines. Osteoarthr. Cartil..

[138] Bruyere O., Cooper C., Pelletier J.P., Maheu E., Rannou F., Branco J., Luisa Brandi M., Kanis J.A., Altman R.D., Hochberg M.C. (2016). A consensus statement on the European Society for Clinical and Economic Aspects of Osteoporosis and Osteoarthritis (ESCEO) algorithm for the management of knee osteoarthritis-From evidence-based medicine to the real-life setting. Semin. Arthritis Rheum..

[139] Watt F.E., Corp N., Kingsbury S.R., Frobell R., Englund M., Felson D.T., Levesque M., Majumdar S., Wilson C., Beard D.J. (2019). Towards prevention of post-traumatic osteoarthritis: Report from an international expert working group on considerations for the design and conduct of interventional studies following acute knee injury. Osteoarthr. Cartil..

[140] Petrigna L., Roggio F., Trovato B., Zanghi M., Guglielmino C., Musumeci G. (2022). How physical activity affects knee cartilage and a standard intervention procedure for an exercise program: A systematic review. Healthcare.

[141] del Río E., Vergés J. (2024). Exploring the influence of physical activity on the efficacy of chondroprotective agents for osteoarthritis: The role of diffusion conditions. Med. Hypotheses.

[142] del Río E. (2025). Rethinking osteoarthritis management: Synergistic effects of chronoexercise, circadian rhythm, and chondroprotective agents. Biomedicines.

[143] Editorial (1984). A national health program for the United States: The need for a citizens coalition. J. Public Health Pol..

[144] Editorial (1987). The role of medical schools in the second epidemiologic revolution. J. Public Health Pol..

[145] Baker E.L., Melius J.M., Millar J.D. (1988). Surveillance of occupational illness and injury in the United States: Current perspectives and future directions. J. Public Health Pol..

[146] Griffin L.Y., Albohm M.J., Arendt E.A., Bahr R., Beynnon B.D., Demaio M., Dick R.W., Engebretsen L., Garrett W.E., Hannafin J.A. (2006). Understanding and preventing noncontact anterior cruciate ligament injuries: A review of the Hunt Valley II meeting, January 2005. Am. J. Sports Med..

[147] Bart K., Wilma J.N., Caspar W.L., Johan P.M. (2014). Educational disparities in the burden of disability: Contributions of disease prevalence and disabling impact. Am. J. Public Health.

[148] Chauvin J., Pauls J., Strobl L. (2016). Building codes: An often overlooked determinant of health. J. Public Health Pol..

[149] Cylus J., Papanicolas I., Smith P.C. (2016). Health System Efficiency: How to Make Measurement Matter for Policy and Management.

[150] Sakellariou G., Conaghan P.G., Zhang W., Bijlsma J.W.J., Boyesen P., D’Agostino M.A., Doherty M., Fodor D., Kloppenburg M., Miese F. (2017). EULAR recommendations for the use of imaging in the clinical management of peripheral joint osteoarthritis. Ann. Rheum. Dis..

[151] Fries J.F. (2005). The compression of morbidity. Milbank. Q..

[152] Iolascon G., Migliore A., Beretta G., Bernetti A., Bortolotti R., Celano A., Giarratano A., Marinangeli F., Momoli A., Sebastiani G.D. (2024). Pain management in knee osteoarthritis: Insights from an exploratory online survey of Italian patients and physicians. Healthcare.

[153] Katz J.N., Arant K.R., Loeser R.F. (2021). Diagnosis and treatment of hip and knee osteoarthritis: A review. JAMA.

[154] Alsobhi M., Gmmash A., Aldhabi R., Almaddah M.R., Ameen A., Almotairi F., Basuodan R., Khan F. (2024). Physical therapists’ attitudes, beliefs, and barriers regarding fall screening and prevention among patients with knee osteoarthritis: A cross-sectional study. Healthcare.

[155] Pijls B.G. (2024). Technology assistance in primary total knee replacement: Hype or hope?. Expert Rev. Med. Devices.

[156] Larson H.J. (2018). The biggest pandemic risk? Viral misinformation. Nature.

[157] Mheidly N., Fares J. (2020). Leveraging media and health communication strategies to overcome the COVID-19 infodemic. J. Public Health Pol..

[158] Wainwright T.W., Burgess L.C., Immins T., Middleton R.G. (2020). Self-management of hip osteoarthritis five years after a cycling and education treatment pathway. Healthcare.

[159] O’Connor M.I. (2006). Osteoarthritis of the hip and knee: Sex and gender differences. Orthop. Clin. N. Am..

[160] Prieto-Alhambra D., Judge A., Javaid M.K., Cooper C., Diez-Perez A., Arden N.K. (2014). Incidence and risk factors for clinically diagnosed knee, hip and hand osteoarthritis: Influences of age, gender and osteoarthritis affecting other joints. Ann. Rheum. Dis..

[161] Wallace I.J., Worthington S., Felson D.T., Jurmain R.D., Wren K.T., Maijanen H., Woods R.J., Lieberman D.E. (2017). Knee osteoarthritis has doubled in prevalence since the mid-20th century. Proc. Natl. Acad. Sci. USA.

[162] del Río E. (2024). A novel etiological approach for the development of knee osteoarthritis in sedentary adults. Med. Hypotheses.

[163] Pineda-Escobar S., Matias-Soto J., García-Muñoz C., Martinez-Calderon J. (2025). Protecting athletes: The clinical relevance of meta-analyses on injury prevention programs for sports and musculoskeletal body regions: An overview of systematic reviews with meta-analyses of randomized clinical trials. Healthcare.

[164] Brown T.D., Johnston R.C., Saltzman C.L., Marsh J.L., Buckwalter J.A. (2006). Posttraumatic osteoarthritis: A first estimate of incidence, prevalence, and burden of disease. J. Orthop. Trauma..

[165] Thomas A.C., Hubbard-Turner T., Wikstrom E.A., Palmieri-Smith R.M. (2017). Epidemiology of posttraumatic osteoarthritis. J. Athl. Train..

[166] Robson K., Pope R., Orr R. (2024). Incidence and risk factors for acute articular cartilage tears in military and other occupational settings: A systematic review. Healthcare.

[167] Nakata K., Shino K., Horibe S., Tanaka Y., Toritsuka Y., Nakamura N., Koyanagi M., Yoshikawa H. (2008). Arthroscopic anterior cruciate ligament reconstruction using fresh-frozen bone plug-free allogeneic tendons: 10-year follow-up. Arthroscopy.

[168] Sadoghi P., von Keudell A., Vavken P. (2012). Effectiveness of anterior cruciate ligament injury prevention training programs. J. Bone Jt. Surg. Am..

[169] Yubo M., Yanyan L., Li L., Tao S., Bo L., Lin C. (2017). Clinical efficacy and safety of mesenchymal stem cell transplantation for osteoarthritis treatment: A meta-analysis. PLoS One.

[170] Bennell K.L., Hunter D.J., Paterson K.L. (2017). Platelet-rich plasma for the management of hip and knee osteoarthritis. Curr. Rheumatol. Rep..

[171] Bensa A., Bianco Prevot L., Moraca G., Sangiorgio A., Boffa A., Filardo G. (2025). Corticosteroids, hyaluronic acid, platelet-rich plasma, and cell-based therapies for knee osteoarthritis - literature trends are shifting in the injectable treatments’ evidence: A systematic review and expert opinion. Expert Opin. Biol. Ther..

[172] Moseng T., Vliet Vlieland T.P.M., Battista S., Beckwée D., Boyadzhieva V., Conaghan P.G., Costa D., Doherty M., Finney A.G., Georgiev T. (2024). EULAR recommendations for the non-pharmacological core management of hip and knee osteoarthritis: 2023 update. Ann. Rheum. Dis..

[173] Grønne D.T., Roos E.M., Ibsen R., Kjellberg J., Skou S.T. (2021). Cost-effectiveness of an 8-week supervised education and exercise therapy programme for knee and hip osteoarthritis: A pre-post analysis of 16 255 patients participating in Good Life with osteoArthritis in Denmark (GLA:D). BMJ Open.

[174] World Health Organization Musculoskeletal Conditions: Fact Sheet. 2022. Geneva, Switzerland. https://www.who.int/news-room/fact-sheets/detail/musculoskeletal-conditions.

[175] Walker A., Boaz A., Gibney A., Zambelli Z., Hurley M.V. (2020). Scaling-up an evidence-based intervention for osteoarthritis in real-world settings: A pragmatic evaluation using the RE-AIM framework. Implement. Sci. Commun..

[176] Docking S., Ademi Z., Barton C., Wallis J.A., Harris I.A., de Steiger R., Buchbinder R., Brusco N., Young K., Pazzinatto M.F. (2024). Lifetime cost-effectiveness of structured education and exercise therapy for knee osteoarthritis in Australia. JAMA Netw. Open.

[177] Hallab N., Merritt K., Jacobs J.J. (2001). Metal sensitivity in patients with orthopaedic implants. J. Bone Jt. Surg. Am..

[178] Gessner B.D., Steck T., Woelber E., Tower S.S. (2019). A systematic review of systemic cobaltism after wear or corrosion of chrome-cobalt hip implants. J. Patient Saf..

[179] Zhong Q., Pan X., Chen Y., Lian Q., Gao J., Xu Y., Wang J., Shi Z., Cheng H. (2024). Prosthetic metals: Release, metabolism and toxicity. Int. J. Nanomed..

[180] Williams J.T., Mambu Vangu A., Balu Mabiala H., Bambi Mangungulu H., Tissingh E.K. (2021). Toxicity in the supply chain: Cobalt, orthopaedics, and the Democratic Republic of the Congo. Lancet Planet Health.

[181] Gorban A.N., Pokidysheva L.I., Smirnova E.V., Tyukina T.A. (2011). Law of the minimum paradoxes. Bull. Math. Biol..

[182] (2022). World Population Prospects 2022: Highlights (ST/ESA/SER.A/470).

[183] Yahaya I., Wright T., Babatunde O.O., Corp N., Helliwell T., Dikomitis L., Mallen C.D. (2021). Prevalence of osteoarthritis in lower middle- and low-income countries: A systematic review and meta-analysis. Rheumatol. Int..

[184] Graham S.M., Howard N., Moffat C., Lubega N., Mkandawire N., Harrison W.J. (2019). Total hip arthroplasty in a low-income country: Ten-year outcomes from the National Joint Registry of the Malawi Orthopaedic Association. JB JS Open Access.

[185] Graham S.M., Moffat C., Lubega N., Mkandawire N., Burgess D., Harrison W.J. (2018). Total knee arthroplasty in a low-income country: Short-term outcomes from a National Joint Registry. JB JS Open Access.

[186] Watt T., Charlesworth A., Gershlick B. (2019). Health and care spending and its value, past, present and future. Future Healthc. J..

[187] Hartman M., Martin A.B., Whittle L., Catlin A. (2024). National health care spending in 2022: Growth similar to prepandemic rates. Health Aff..

